# Exposure-Midpoint Temporal Alignment and Risk-Aware Adaptive Feature Tracking for Stereo Visual–Inertial Odometry

**DOI:** 10.3390/s26185849

**Published:** 2026-09-15

**Authors:** Sucheng Yang, Qingqing Liu, Zhihong Zhuang, Xiaofeng Shen

**Affiliations:** 1 School of Electronic and Optical Engineering, Nanjing University of Science and Technology, Nanjing 210094, China; yangsucheng@njust.edu.cn; 2 Department of Electronic and Electrical Engineering, University College London, London WC1E 7JE, UK; qingqing.liu.24@ucl.ac.uk; 3 Applied Technology College, Soochow University, Suzhou 215000, China; fly_sxf3@163.com

**Keywords:** stereo visual–inertial odometry, exposure midpoint, dynamic exposure, auto-exposure, adaptive KLT tracking, camera–IMU synchronization

## Abstract

Visual–inertial odometry (VIO) on industrial stereo camera–IMU platforms suffers from frame-dependent timing errors and degraded feature tracking under auto-exposure, auto-gain adjustment, and rapid motion. This paper presents Hardware-Synchronized Exposure-State-Aware VINS-Fusion (HS-ES-VINS), a hardware-synchronized, exposure-aware extension of VINS-Fusion. The effective sampling time of each global-shutter image is reconstructed at the exposure midpoint using synchronized trigger timestamps, the camera debounce delay, and the frame-wise exposure duration. An exposure–gain–motion risk model adaptively adjusts feature detection criteria, Kanade–Lucas–Tomasi (KLT) tracking parameters, and correspondence filtering, while IMU-integrated rotation supplies initial predictions for feature tracking. On hardware-synchronized indoor and outdoor datasets with independent ground truth, controlled experiments show that the exposure-midpoint timestamp removes the frame-dependent exposure component of the camera–IMU offset that neither fixed nor online scalar compensation can remove, reducing the ATE RMSE by 28.6% (fixed offset) and 22.7% (online estimation) relative to the exposure-start timestamp under 20 ms low-light exposure. Compared with baseline VINS-Fusion, the complete adaptive front end reduces the ATE RMSE by 16.8% and 12.9% on indoor multi-floor and outdoor cycling sequences, and the risk score explains frame-wise tracking quality (correlation of 0.587 with the inlier ratio). The front end runs faster than the baseline (17.1 ms per frame), confirming real-time operation and improved robustness against variable illumination and rapid motion.

## 1. Introduction

Visual–inertial odometry (VIO) fuses camera observations with inertial measurement unit (IMU) data for six-degree-of-freedom pose estimation in GNSS-denied environments, including indoor navigation, underground spaces, mobile mapping, and urban canyons. Optimization-based systems such as VINS-Fusion extend the tightly coupled sliding-window framework of VINS-Mono to monocular, stereo, and multi-sensor configurations, balancing accuracy, robustness, and real-time performance [1]. However, when transferred from public datasets and laboratories to industrial stereo camera–IMU platforms, performance depends not only on back-end optimization but also on the physical meaning of sensor timestamps and the reliability of front-end features. This is especially important for global-shutter cameras operating under auto-exposure (AE) and auto-gain (AG), where the offset between the assigned timestamp and actual acquisition time varies by frame, while exposure, gain, and motion jointly degrade local image texture.

In practical robotic systems, images and IMU data are acquired and transmitted through data acquisition cards, industrial computers, and software middleware. An image timestamp may indicate trigger arrival, driver callback, readout completion, transmission completion, or host reception rather than the physical capture instant. Even with hardware triggering or timestamping, it remains necessary to determine whether the timestamp refers to exposure start, midpoint, or end, or a data-ready event. A global-shutter frame integrates photons over a finite exposure interval, so its effective measurement time is generally the exposure midpoint rather than the trigger time. Under AE, frame-dependent exposure duration causes the offset between these times to vary. A fixed camera–IMU delay obtained by offline calibration therefore produces an inconsistency between visual observations and IMU preintegration intervals. During rapid rotation, strong vibration, or long-exposure low-light motion, rotational integration and reprojection geometry amplify this error, destabilizing feature tracking and increasing pose drift. These two deficiencies have quantifiable consequences. In the controlled experiments presented in Section 4, shifting the frame timestamps by 10ms—the half-exposure offset that arises when auto-exposure sets the exposure time to 20ms in low light—increases the ATE RMSE of VINS-Fusion by up to 40%. Under challenging illumination conditions, feature tracking degrades even more directly: in our experiments, frames classified as high risk exhibit an inlier ratio of 55.6% and a mean feature-track length of only 10.4 frames, compared with 80.5% and 53.3 frames under low risk (Section 4.2.3). Field studies of outdoor visual SLAM similarly show that ambient illumination significantly affects localization accuracy, and that a minimum illuminance of 434 lux is required for a consistent performance [2].

The VINS front end mainly uses Shi–Tomasi corner detection and Kanade–Lucas–Tomasi (KLT) optical flow. Although efficient, it assumes sufficient local texture and approximate brightness constancy between consecutive frames. In industrial scenes, AE and AG continuously alter photometric conditions: long exposure causes motion blur, high gain amplifies sensor noise, and abrupt exposure or gain changes violate KLT intensity consistency. Direct visual odometry and photometric calibration studies have shown that exposure duration, camera response, and lens vignetting strongly affect odometry accuracy [3,4]. Auto-exposure research further links exposure control to image quality and localization robustness under difficult illumination conditions [5]. Nevertheless, most feature-based VIO systems treat exposure and gain only as metadata rather than using them to assess feature extraction, optical-flow tracking, and outlier reliability.

To address these limitations, this paper proposes Hardware-Synchronized Exposure-State-Aware VINS-Fusion (HS-ES-VINS), which extends VINS-Fusion through frame-wise temporal alignment and an exposure-, gain-, and motion-aware front end. The main contributions are as follows:

1. We propose a hardware-synchronized frame-wise temporal alignment model for stereo VINS-Fusion. Using synchronized IMU trigger timestamps, fixed camera trigger debounce delay, and per-frame exposure duration, the model reconstructs the effective image sampling time at the exposure midpoint. The camera–IMU offset is therefore represented not only as a fixed offline-calibrated parameter but also as an observable frame-varying quantity determined by exposure state.
2. We develop a joint exposure–gain–motion risk model for per-frame visual reliability. It combines exposure duration, analog gain, inter-frame exposure and gain changes, and angular motion obtained by integrating gyroscope measurements over the exposure interval. Unlike exposure-only models, it captures pixel-level blur risk caused by the interaction between long exposure and actual camera motion.
3. We design an adaptive KLT tracking strategy driven by the joint risk score. It dynamically adjusts the Shi–Tomasi quality threshold, optical-flow window size, pyramid levels, and forward–backward consistency threshold. Feature correspondences are further screened using local saturation ratio, zero-mean normalized cross-correlation (ZNCC), and Lucas–Kanade (LK) photometric error. IMU rotational integration between adjacent exposure midpoints also supplies an initial warp prediction, improving tracking under large rotations and long-exposure low-light conditions.

The remainder of this paper is organized as follows. Section 2 reviews VIO, camera–IMU temporal calibration, hardware synchronization, and photometric robustness. Section 3 presents the exposure-midpoint alignment model and exposure-, gain-, and motion-aware front-end method. Section 4 describes the experimental platform and comparative results. Section 5 concludes the paper.

## 2. Related Work

Visual–inertial odometry (VIO) provides high-rate pose estimation for robotic platforms, unmanned aerial vehicles, and augmented reality systems. Filtering-based methods such as MSCKF have established an efficient probabilistic framework for real-time visual–inertial state estimation [6]. Sliding-window optimization methods, including OKVIS and VINS-Mono, jointly optimize camera poses, IMU states, feature observations, and sensor parameters through nonlinear least squares, improving accuracy and robustness [1,7]. ORB-SLAM3 further supports monocular, stereo, RGB-D, and visual–inertial configurations with enhanced map reuse [8]. Recent front-end improvements include offloading optical flow to global-shutter sensors to reduce VINS-Mono latency and processor load [9], and combining point and structural line features for complex man-made environments [10]. However, these pipelines usually treat image timestamps as exact measurement instants and assume that geometrically retained features have comparable reliability, assumptions that may fail for industrial stereo cameras using auto-exposure and auto-gain.

Camera–IMU temporal misalignment is a major source of VIO inconsistency. Kalibr and iKalibr jointly estimate temporal offsets and spatial extrinsics [11,12], while Rehder and Siegwart analyze the effects of IMU intrinsics, inter-axis misalignment, and camera models on calibration accuracy [13]. Online methods incorporate temporal offset into monocular VIO [14], augment EKF states with unknown delay and refine initial attitude using complementary filtering [15], or use continuous-time trajectories to separate dynamic IMU excitation from static visual observations, reducing the trade-off between motion blur and insufficient excitation [16]. These studies show that misalignment disrupts both visual reprojection and IMU-preintegration residuals. Nevertheless, most methods model delay as a constant or slowly varying scalar and do not distinguish trigger assertion, exposure onset, exposure midpoint, readout completion, driver timestamping, and host reception. Under auto-exposure, such models cannot capture frame-dependent changes in effective measurement time caused by varying exposure duration.

Hardware synchronization reduces system-level timestamp uncertainty and underpins many camera–IMU systems [17,18,19]. Its importance is reflected in synchronized benchmarks: EuRoC provides stereo images, IMU data, and high-precision ground truth [20]; TUM VI adds high-resolution images, photometric calibration, and high-dynamic-range sequences [21]; and TUM-VIE combines stereo events, grayscale images, and IMU measurements with synchronized timestamps [22]. However, residual latency and jitter may persist after synchronization [23,24], and a hardware timestamp alone does not identify its physical reference event. Global-shutter frames share one exposure interval, whereas rolling-shutter rows have distinct exposure midpoints. Rolling-shutter VO and VIO studies show that ignoring this time-varying image geometry reduces accuracy and robustness [25,26]. Hardware timestamps therefore require explicit exposure-process modeling for physical-event-level alignment.

A digital image integrates scene irradiance over a finite exposure interval rather than sampling an instant [27]. For global-shutter cameras, the exposure midpoint is the effective frame time; for rolling-shutter cameras, row-dependent midpoints create geometric distortion during fast motion [28]. IMU preintegration requires accurate temporal boundaries between visual frames [29]; therefore, timestamps displaced from the true exposure midpoint bias the integration interval. Such errors may be masked under mild motion by visual noise, robust losses, or iterative correction, but millisecond offsets become significant during rapid rotation, acceleration, deceleration, or vibration because rotational integration and reprojection geometry amplify them [30,31]. Luan et al. further use IMU measurements to estimate image-point blur trajectories over the exposure interval and perform hierarchical screening and selective restoration, showing that inertial motion can quantify front-end image degradation [32].

Photometric research likewise demonstrates the importance of exposure state. DSO explicitly models camera response, vignetting, and exposure duration [3]; stereo direct VO estimates inter-frame exposure ratios and vignetting for gamma-like response curves [4]; and auto-exposure studies link exposure control to localization robustness under difficult illumination conditions [5]. Motion-blur research shows that low-light, long-exposure imaging under rapid motion severely degrades VO, while modeling image-formation trajectories over the exposure window improves robustness [33]. Stereo direct sparse VIO jointly optimizes photometric and inertial residuals and identifies dynamic illumination as a key challenge [34]; UAV-oriented engineering methods further improve stability under variable illumination, weak texture, and blur through multi-camera configurations and adaptive processing [35]. Event cameras provide high temporal resolution, low latency, and wide dynamic range: event accumulation frames can support tracking in conventional images [36], while ESVO2 combines stereo event-based direct estimation with IMU preintegration [37]. However, these studies primarily address direct photometric modeling, calibration, exposure control, deblurring, or event vision. They do not directly target feature-based KLT tracking in conventional industrial frame cameras or jointly exploit exposure duration, analog gain, and synchronized IMU motion for feature extraction, optical-flow adaptation, and outlier rejection.

Overall, existing VIO methods achieve high accuracy but usually assign one timestamp to an entire frame and retain fixed front-end parameters despite changing illumination and motion. Temporal calibration seldom links estimated delay to the per-frame exposure midpoint, while hardware synchronization cannot itself map timestamps to physical exposure events. Photometric, auto-exposure, and blur-aware studies establish the importance of exposure state but have not fully integrated it into VINS-style KLT pipelines. RISE-VIO recently used IMU priors to filter correspondences under weak texture, abrupt illumination changes, and high outlier rates [38], but did not explicitly include per-frame exposure duration or analog gain. This work therefore addresses industrial global-shutter stereo cameras operating with auto-exposure and auto-gain by jointly using synchronized timestamps, per-frame exposure and gain metadata, and IMU motion to improve camera–IMU temporal alignment and front-end tracking robustness.

## 3. Methodology

### 3.1. System Overview

**Figure 1:**
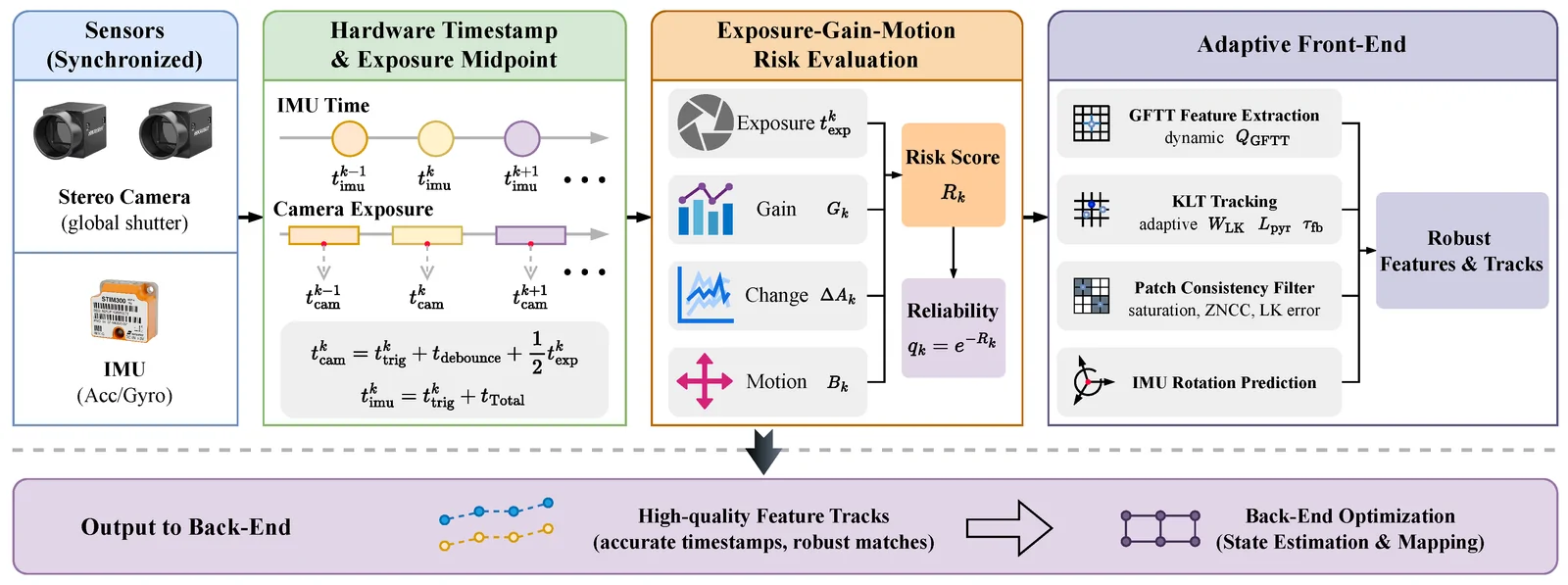
Hardware-synchronized timing and risk-aware adaptive front end for robust visual–inertial feature tracking. White arrows denote the sensor data flow, and black arrows denote the data processing flow.

This section addresses two critical limitations of the VINS-Fusion front end: inaccurate temporal alignment and frequent feature tracking failures under challenging illumination conditions, auto-exposure adjustment, and high-dynamic motion. An adaptive front-end framework is proposed by integrating hardware-level time synchronization with exposure-, gain-, and motion-aware mechanisms. As illustrated in Figure 1, the limitations of the baseline front-end pipeline are first analyzed from three dimensions: fixed image timestamps, the local brightness constancy assumption, and invariant tracking parameters. A hardware-synchronized multi-sensor system is then employed to derive the exposure midpoint of each frame from the hardware trigger timestamp and frame-wise exposure duration, thereby achieving exposure-midpoint temporal alignment between camera observations and IMU measurements. Building on the synchronized measurements, visual observation risk and reliability metrics are constructed by jointly considering exposure time, analog gain, inter-frame imaging state variation, and angular motion over the exposure interval. These metrics are further utilized to adaptively tune the good features to track (GFTT) feature extraction threshold and KLT optical flow tracking parameters. In addition, unreliable feature correspondences are progressively filtered via a stepwise screening procedure based on local patch saturation, ZNCC consistency, and LK photometric residuals, while IMU-derived rotation prediction provides initial estimates for KLT tracking. The resulting enhanced front end generates precisely time-aligned feature observations with reliable inter-frame correspondences across consecutive frames, providing stable visual constraints for subsequent state estimation and mapping processes.

### 3.2. Baseline VINS-Fusion Front End

**Figure 2:**
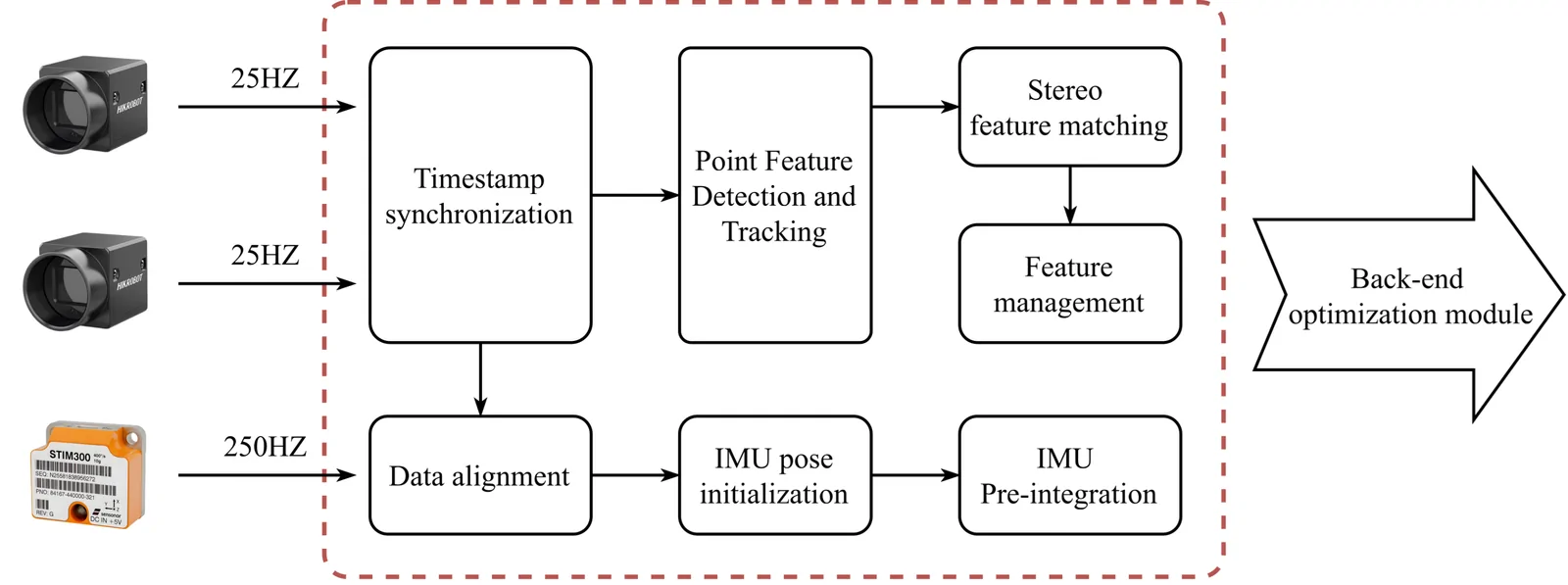
Visual–inertial front-end pipeline with timestamp synchronization, stereo feature tracking, and IMU preintegration.

The VINS-Fusion front end mainly extracts, tracks, and maintains stable visual feature points from sequential stereo frames to supply valid visual observation constraints for sliding-window optimization at the back end. As illustrated in Figure 2, the entire processing pipeline consists of five core stages: sensor timestamp synchronization and data alignment, feature detection, optical flow tracking, stereo matching, and feature management. To begin with, the system receives left and right stereo frames and preliminarily associates each image with buffered IMU measurements based on corresponding timestamps. Next, Shi–Tomasi corner detection [39] is used to extract feature points with prominent texture responses from the raw images. A grid-based uniform sampling strategy is further adopted to regularize the spatial distribution of feature points across the image plane. This avoids dense clustering within small local areas and strengthens the geometric stability of the resulting visual constraints.

Across temporally successive frames, the VINS-Fusion front end adopts the KLT optical flow algorithm [40] to track persistent feature points. Rooted in the local brightness constancy hypothesis for adjacent images, this algorithm iteratively optimizes to compute the coordinates of tracked features within the current frame. To enhance tracking robustness, a forward–backward optical flow consistency test is integrated to filter out features that exhibit jittery tracking behavior or substantial positional drift. When operating with stereo inputs, the front end additionally establishes feature correspondences between the left and right camera views. Mismatched feature pairs are eliminated by imposing epipolar constraints, disparity range bounds, and essential matrix geometric constraints. Upon completing all screening procedures, the remaining valid feature points are aggregated into inter-frame feature tracks and transmitted to the back end to support state estimation, 3D point triangulation, and reprojection error minimization.

The native front-end pipeline of VINS-Fusion inherently assumes that each assigned image timestamp precisely corresponds to the true sampling instant of the frame. It further presumes that brightness fluctuations and exposure discrepancies across successive frames will not substantially violate the core hypotheses underpinning optical flow tracking. Nevertheless, real embedded hardware platforms introduce multiple non-ideal factors that distort each frame’s actual imaging timing, including trigger latency, variable exposure duration, data transmission lag, and timestamping bias generated on the host processor. Moreover, the camera’s auto-exposure mechanism inevitably induces frame-wise fluctuations in exposure length and overall image quality. When the sensor platform undergoes rapid rotational or translational motion alongside drastic illumination shifts, captured frames are prone to motion blur, attenuated feature intensities, and outright optical flow matching breakdowns.

### 3.3. Hardware-Provided Temporal Model

For the temporal synchronization between IMU and camera data, conventional approaches use periodic signals to trigger the IMU and camera simultaneously, followed by data acquisition through ROS [18]. However, such methods have several limitations. First, they require the IMU to support external triggering. Second, the timestamps of IMU and camera data are based on the time when the system receives the measurements, which introduces temporal errors caused by internal sensor latency, data transmission delays, and operating system scheduling. Consequently, even with hardware triggering using synchronization signals, the temporal correspondence between IMU and camera measurements remains weak. Performing offline temporal calibration and online time offset evaluation on such data may lead to unreliable algorithmic results. Therefore, high-quality synchronized data is an essential foundation before conducting algorithmic research.

**Figure 3:**
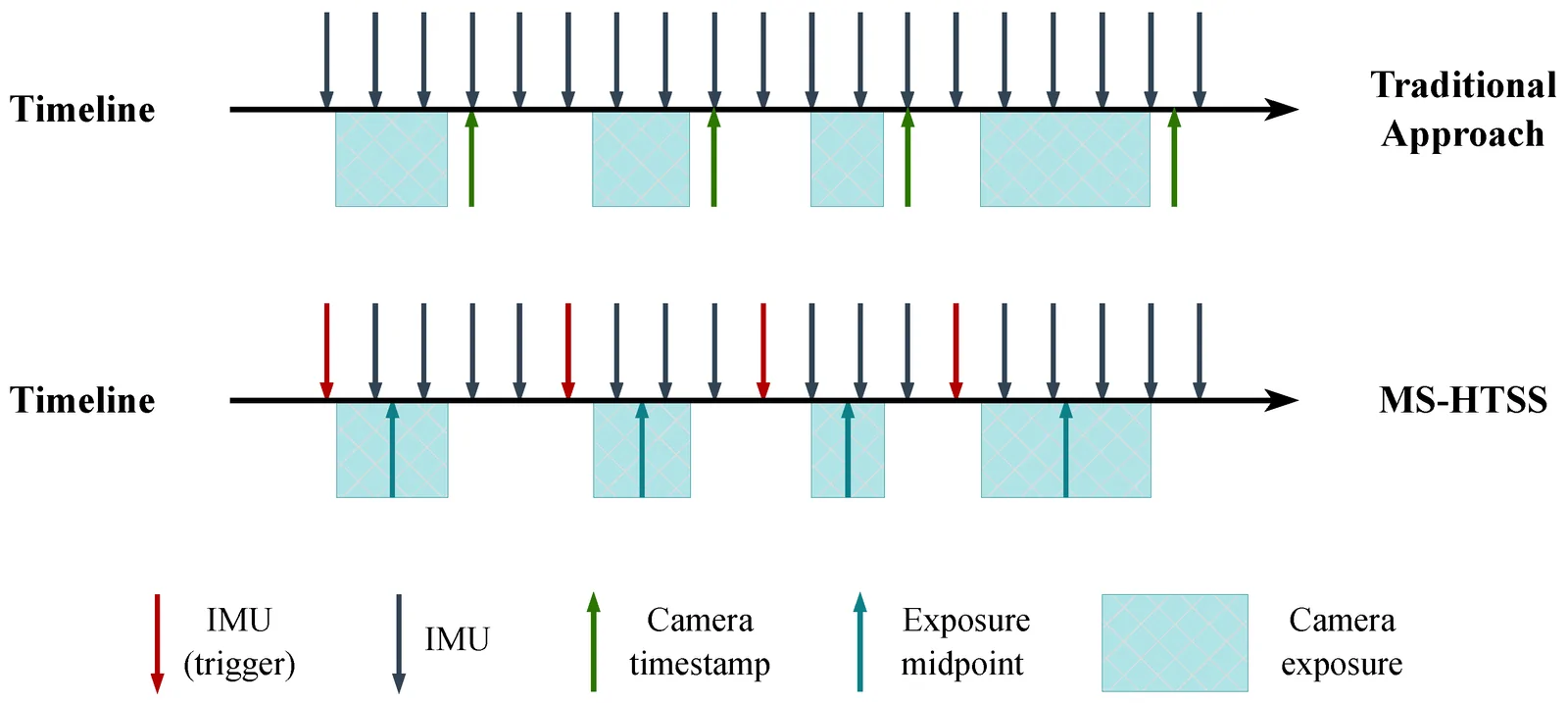
Hardware temporal alignment of IMU and camera data based on MS-HTSS.

As shown in Figure 3, our previous work, the Multi-Sensor Hardware Time Synchronization System (MS-HTSS), achieves synchronized acquisition of non-triggered IMU data and triggered global-shutter camera data under a unified time reference [41]. Compared with other data acquisition approaches, MS-HTSS provides accurate hardware timestamps for both IMU and camera measurements, even when the camera operates in auto-exposure mode. Moreover, it can associate each camera exposure trigger event with the corresponding precise IMU sample, which provides a critical basis for aligning the temporal boundaries between image and IMU data.

Let $t_{\mathrm{trig}}^{k}$ denote the timestamp of the trigger event for the *k*-th image, $t_{\mathrm{debounce}}$ denote the fixed internal trigger debounce time of the camera, which is 50μs, and $t_{\mathrm{exposure}}^{k}$ denote the exposure time of the *k*-th image. Then, the effective sampling time of the *k*-th image can be expressed as

(1) $$t_{\mathrm{cam}}^{k}=t_{\mathrm{trig}}^{k}+t_{\mathrm{debounce}}+\frac{1}{2}t_{\mathrm{exposure}}^{k}$$

In the proposed platform, the frame-wise exposure duration $t_{\mathrm{exposure}}^{k}$ is obtained from the camera driver rather than estimated: the industrial camera reports the exposure time and analog gain actually applied to each frame as image metadata, and MS-HTSS delivers this metadata together with the hardware trigger timestamp of the corresponding frame on the shared clock. Since each trigger event initiates exactly one exposure interval of the global-shutter sensor, the reported exposure duration is uniquely associated with its image without any software-side inference. Two validations support this association. First, under low light, AE sets the exposure time to a constant value of 20ms, so the derived midpoint offset is exactly 10ms, and the controlled experiment presented in Section 4.1 shows that the trajectory accuracy is indeed optimized precisely at this offset. Second, under normal illumination, the reported exposure time varies frame by frame between 0.4 and 6.4ms, yet the online estimated time offset under the midpoint reference remains nearly identical across the two illumination conditions (14.979 vs. 15.045ms), which would not hold if the reported exposure duration were inconsistent with the actual integration window. Residual discrepancies, e.g., between the reported exposure value and the actual integration window, are summarized by $\delta t_{\mathrm{cam}}$ in Equation (3).

**Figure 4:**
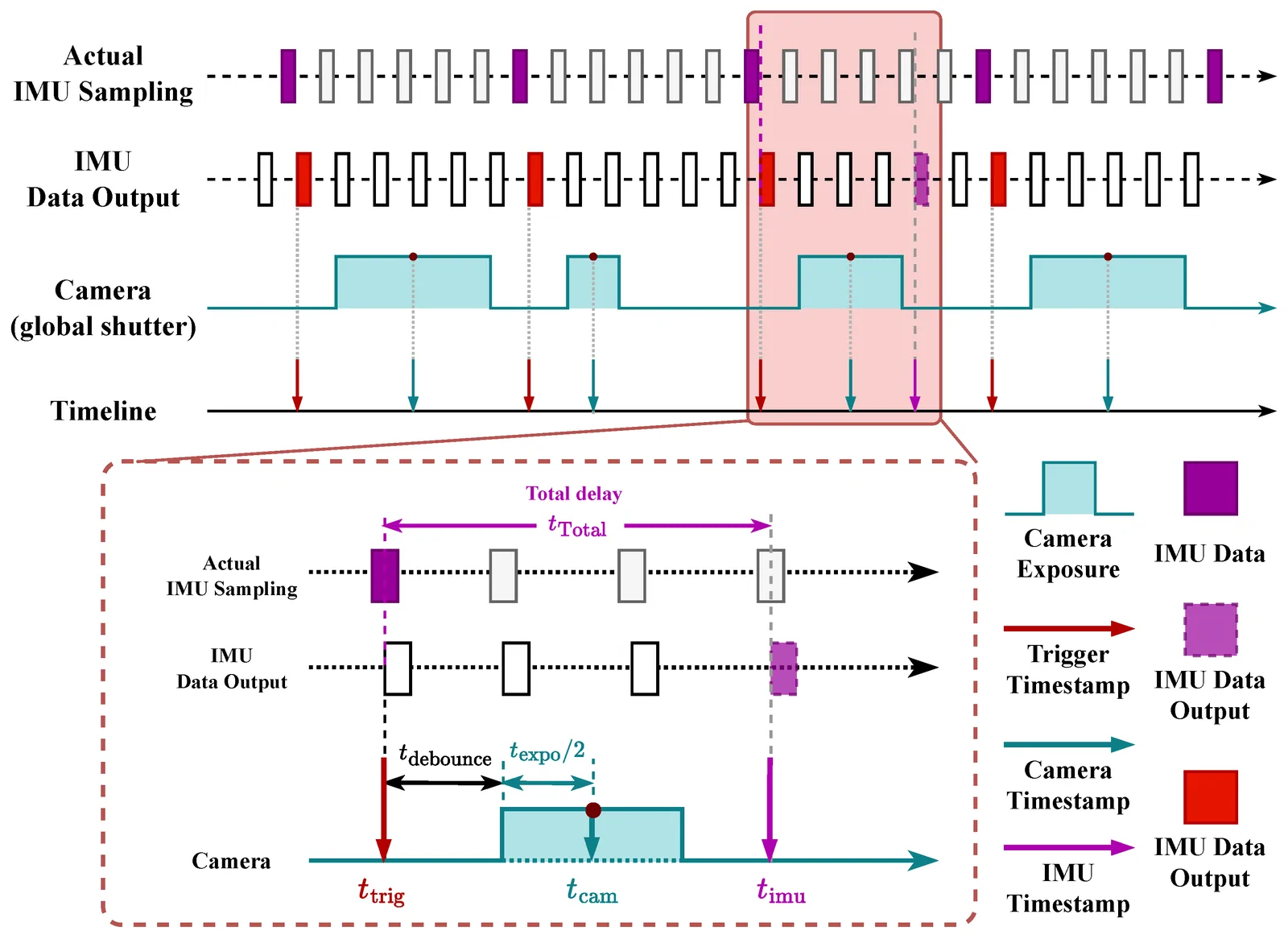
Hardware timestamp annotation during IMU-triggered global-shutter camera exposure process. The red box marks the exposure interval, the gray dashed line denotes the timestamp, and the purple dotted line denotes the IMU sampling events.

This model indicates that the effective image sampling time is not always equal to the trigger time, but varies across frames according to the actual camera exposure time. We further analyze the sources of temporal errors between the camera and IMU. Figure 4 illustrates the complete data acquisition process.

**Figure 5:**
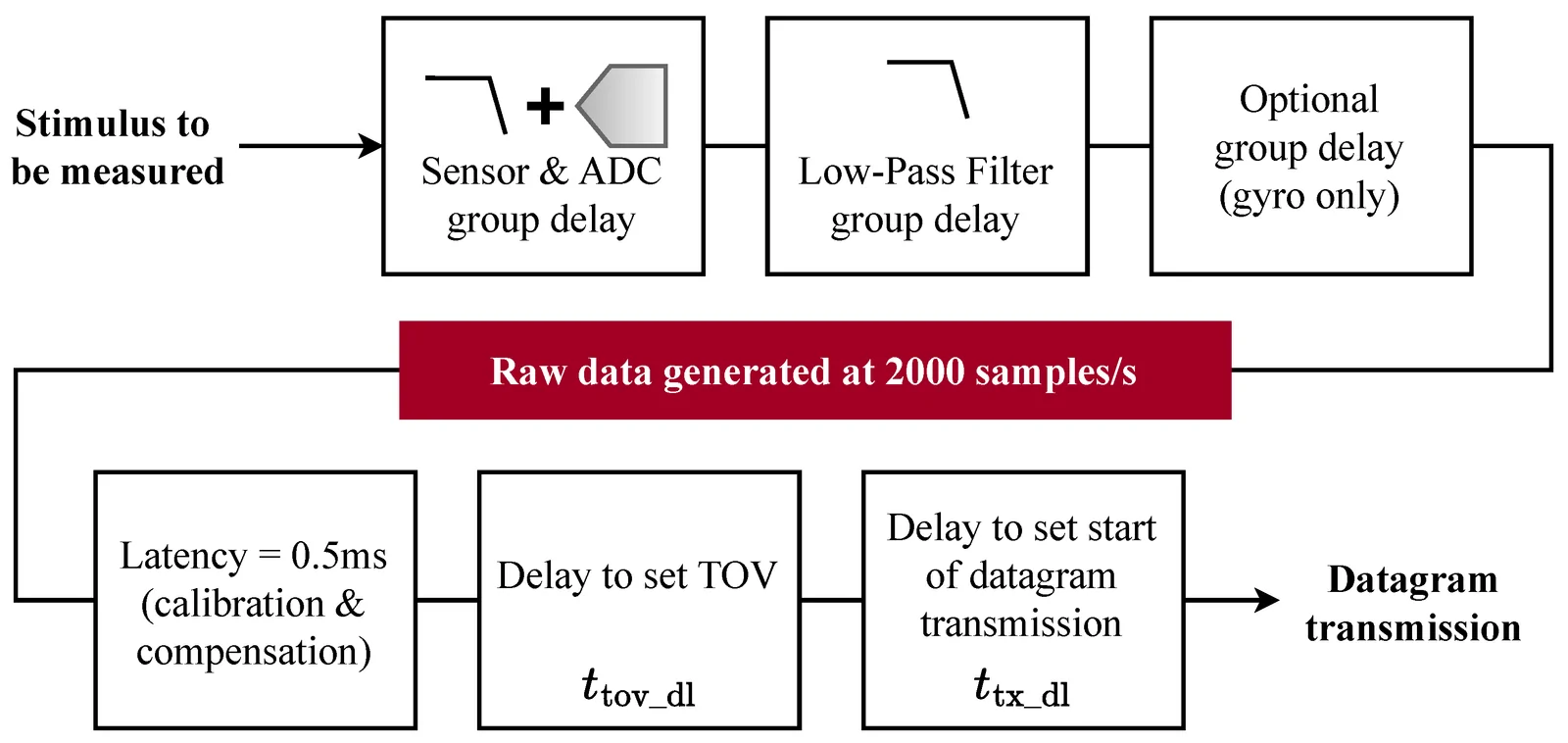
Time delays when sampling rate is not set to “External Trigger”.

MS-HTSS timestamps each IMU sample at the data output time rather than the physical sampling time. The difference between these two times corresponds to $t_{\mathrm{Total}}$ in Figure 5. The STIM300 inertial measurement unit used in our platform introduces internal delays associated with analog-to-digital conversion, low-pass filtering, and serial data output. The detailed processing pipeline is illustrated in Figure 5.

The total time delay model of the STIM300 operating in non-triggered mode is given by [42]

(2) $$\begin{array}{cc} t_{\mathrm{Total}} & =t_{\mathrm{Sensor}\&\mathrm{ADC}}\\ \; & +1.5\;\mathrm{ms}\cdot \mathrm{integer}[\frac{262\mathrm{Hz}}{\mathrm{LowPassFilter}}+0.5]\\ \; & +t_{\mathrm{LPF}\_\mathrm{DelayOffset}}+t_{\mathrm{OptionalDelay}}+t_{\mathrm{tx}\_\mathrm{dl}}+t_{\mathrm{tov}\_\mathrm{dl}}\\ \; & +\frac{0.5\mathrm{ms}\cdot \sum_{n=1}^{\#samples}n}{\#samples}\\ \; & +\frac{\#datagram\_bytes\cdot \left(1+8+\#parity\_bit+\#stop\_bits\right)}{bit\_rate}, \end{array}$$

Here, #samples denotes the number of samples aggregated in each output. For incremental or averaged output units, #samples=2000/SampleRate. In our platform, the output units are angular rate and acceleration, without incremental or averaged output, and each output contains only the latest sample. Therefore, #samples=1, resulting in a corresponding delay term of 0.5ms. Under the configuration used in our platform, the low-pass filters of both the gyroscope and accelerometer channels are set to 33Hz, the output units are neither incremental nor averaged, and the delayed-output option is disabled for the gyroscope. Substituting these settings into the delay model, the total delays from physical sampling to the start of data output for each channel in non-triggered mode are summarized in Table 1. The total delay is approximately 13.2ms for the gyroscope channel and 17.6ms for the accelerometer channel with the 10g range.

This delay is constant, independent of the exposure time and camera configuration, and can be consistently reproduced under all illumination conditions of our platform. The offline calibration using Kalibr converges to 13.828ms. Meanwhile, the online time offset estimation in VINS converges to 14.979ms and 15.045ms under low-light and normal illumination when the frame timestamp is referenced to the exposure midpoint, and shifts correspondingly when other timestamp references are used (see the controlled experiments presented in Section 4.1). Both results are on the same order of magnitude as the gyroscope channel delay calculated from the above model (13.2ms). Since the observability of the time offset is mainly provided by rotational motion, the single scalar offset estimated by the estimator is attracted to the vicinity of the gyroscope channel delay. The accelerometer channel delay (17.6ms) differs from the gyroscope delay by approximately 4.3ms, and no single scalar offset can accurately compensate for both channels simultaneously. This inter-channel residual, together with the frequency-dependent group delay introduced by the low-pass filters, constitutes the remaining source of temporal misalignment.

It is worth noting that MS-HTSS does not compensate for $t_{\mathrm{Total}}$, nor is such compensation necessary. A constant delay is exactly the type of offset that fixed or online offset estimators are designed to absorb. Eliminating this delay through hardware would require reconstructing the physical sampling time from the external trigger timestamp and the per-sample latency field contained in the data packet, followed by re-timestamping each IMU sample. This process exceeds the design scope of the hardware system, which is intended to eliminate the exposure-related frame-wise timing component, and does not provide additional accuracy benefits. Therefore, the responsibility boundary of MS-HTSS is defined as follows: it reconstructs the image exposure-midpoint timestamp on a frame-by-frame basis and removes the exposure-related timing component on the image side, while the constant IMU-side delay $t_{\mathrm{Total}}$ is left to be absorbed by the estimator.

The frame-wise component on the image side is the offset between the trigger time and the effective sampling time, which consists of the fixed debounce time and half of the exposure time:

(3) $$\Delta t_{\mathrm{IC}}^{k}=t_{\mathrm{debounce}}+\frac{1}{2}t_{\mathrm{exposure}}^{k}+\delta t_{\mathrm{cam}},$$

Here, $\delta t_{\mathrm{cam}}$ only summarizes the residual errors on the camera side, including the discrepancy between the exposure value reported by the camera driver and the actual integration window, as well as the instantaneous deviation introduced during the AE adjustment process. $\Delta t_{\mathrm{IC}}^{k}$ varies across frames with the camera exposure time. Under low-light conditions, when the exposure time is set by AE to 20ms, its value approaches 10.05ms. Under normal illumination, when the exposure time fluctuates between 0.4 and 6.4ms, it becomes a frame-dependent variable ranging from approximately 0.25 to 3.25ms.

In this section, exposure-midpoint temporal alignment contributes to using MS-HTSS to reconstruct the exposure midpoint of each frame at the hardware level and using the midpoint timestamp defined in Equation (1) as the frame timestamp. In this way, the frame-wise exposure-related component is directly removed from the data.

### 3.4. Exposure–Gain–Motion-Aware Patch Reliability Front End

The baseline VINS-Fusion front-end pipeline predominantly adopts the KLT optical flow algorithm to track feature points across consecutive frames. The core assumption underlying KLT optical flow is the local brightness constancy constraint, which posits that the local image patch corresponding to the same spatial point maintains an approximately invariant intensity distribution across adjacent frames. This constraint is mathematically formulated as

(4) $$I_{k}\left(x+u\right)\approx I_{k-1}\left(x\right),$$

where $I_{k-1}$ and $I_{k}$ denote the intensity images of frame k−1 and frame k, respectively, x represents the feature coordinate in the previous frame, and u denotes the optical flow displacement of the feature point across two consecutive frames. This assumption generally holds under stable illumination, minor variations in exposure parameters, and smooth camera motion. However, under highly dynamic illumination or low-light scenarios, industrial cameras automatically tune exposure duration and analog gain based on the average brightness of the full frame or a predefined region of interest. Variations in exposure duration directly alter overall image brightness and induce motion blur during rapid camera motion. Elevating analog gain enhances image brightness but concurrently amplifies sensor noise. Consequently, long exposure, high analog gain, and abrupt exposure fluctuations across consecutive frames violate the intensity consistency of local image patches, leading to unstable feature corners in texture-sparse regions, high-contrast edges, and reflective surfaces, which ultimately degrades the reliability of KLT tracking.

To improve the robustness of the VINS-Fusion front end under challenging illumination conditions, this section extends the original KLT-based front-end pipeline by incorporating camera exposure parameters, analog gain, and IMU motion information to construct an adaptive front end with exposure-, gain-, and motion-aware capabilities. Instead of directly masking image regions, the proposed method improves the reliability of visual features and tracking results through frame-level risk assessment, adaptive adjustment of the GFTT feature detection threshold, adaptive KLT parameter selection, patch-level photometric consistency validation, and IMU-based rotation prediction.

#### 3.4.1. Exposure–Gain–Motion-Based State Modeling and Joint Observation Risk Assessment

For the *k*-th image frame, the joint exposure–gain state is defined as

(5) $$A_{k}=\log\left(t_{\exp}^{k}+\epsilon_{t}\right)+\log\left(G_{k}+\epsilon_{g}\right),$$

where $t_{\exp}^{k}$ denotes the exposure time of the k-th frame, $G_{k}$ denotes the analog gain in its linear form, and $\epsilon_{t}$ and $\epsilon_{g}$ are small positive constants introduced to avoid singularities in the logarithmic operation. When the camera reports gain values in decibels (dB), they are first converted to their linear form as

(6) $$G_{k}=10^{g_{k}/20},$$

where $g_{k}$ denotes the gain value reported by the camera in decibels (dB). Since both exposure time and analog gain exert an approximately multiplicative effect on image brightness, the logarithmic transformation converts them into an additive representation, which facilitates the description of photometric state variations jointly induced by auto-exposure and auto-gain control.

The exposure–gain variation between two consecutive frames is defined as

(7) $$\Delta A_{k}=\left|A_{k}-A_{k-1}\right|,$$

where $\Delta A_{k}$ characterizes the inter-frame photometric perturbation induced by auto-exposure and auto-analog-gain control. A larger $\Delta A_{k}$ indicates a more pronounced variation in imaging state across consecutive frames, which increases the likelihood of violating the brightness constancy assumption required by KLT.

In addition to exposure and gain variations, camera motion over the exposure interval constitutes another critical factor affecting the stability of the visual front-end pipeline. As described in Section 3.3, hardware timestamp synchronization and exposure-midpoint compensation are performed in advance, enabling each image frame to be accurately associated with its actual exposure interval along the IMU time axis. Let the exposure interval of the k-th image frame be defined as

(8) $$\left[t_{\mathrm{start}}^{k},\;t_{\mathrm{start}}^{k}+t_{\exp}^{k}\right],$$

where $t_{\mathrm{start}}^{k}$ denotes the exposure start timestamp of the k-th image frame. Using gyroscope measurements acquired via the hardware-synchronized timing system, the angular velocity magnitude can be integrated over the exposure interval to quantify the angular motion intensity during exposure as

(9) $$\theta_{\exp}^{k}=\int_{t_{\mathrm{start}}^{k}}^{t_{\mathrm{start}}^{k}+t_{\exp}^{k}}∥\omega \left(t\right)∥dt,$$

where ω(t) denotes the angular velocity measured by the IMU at time t, $∥\omega \left(t\right)∥$ denotes its scalar magnitude, and $\theta_{\exp}^{k}$ represents the accumulated rotational angle of the camera over the exposure interval. This metric jointly captures the combined effect of exposure duration and actual motion intensity within the exposure window. Furthermore, this angular motion intensity can be approximately mapped to a pixel-level motion-blur risk as

(10) $$B_{k}=f\cdot \theta_{\exp}^{k},$$

where *f* denotes the camera focal length measured in pixels. $B_{k}$ represents the potential pixel displacement on the image plane induced by camera rotation over the exposure interval. Compared with using exposure duration alone, $B_{k}$ provides a more accurate characterization of motion-blur risk, since prolonged exposure does not necessarily result in image blurring. Significant motion blur arises only when long exposure coincides with substantial camera motion within the exposure window. After obtaining the exposure duration, gain variation, and IMU-derived motion blur information, four normalized risk components are defined as follows:

(11) $$\begin{array}{cc} R_{\exp}^{k} & =\frac{t_{\exp}^{k}}{t_{\mathrm{ref}}}\\ R_{\mathrm{gain}}^{k} & =\frac{\log\left(G_{k}+\epsilon_{g}\right)}{\log\left(G_{\mathrm{ref}}+\epsilon_{g}\right)}\\ R_{\mathrm{change}}^{k} & =\frac{\Delta A_{k}}{\Delta A_{\mathrm{ref}}}\\ R_{\mathrm{motion}}^{k} & =\frac{B_{k}}{B_{\mathrm{ref}}} \end{array}$$

where $R_{\exp}^{k}$ denotes the exposure-time risk, and $t_{\mathrm{ref}}$ is the reference exposure duration; $R_{\mathrm{gain}}^{k}$ denotes the gain-related risk, and $G_{\mathrm{ref}}$ is the reference linear gain; $R_{\mathrm{change}}^{k}$ denotes the inter-frame exposure–gain variation risk, and $\Delta A_{\mathrm{ref}}$ is the reference threshold for acceptable exposure–gain variation; and $R_{\mathrm{motion}}^{k}$ denotes the motion-blur risk over the exposure interval, and $B_{\mathrm{ref}}$ is the reference pixel blur threshold. The normalization procedure maps these heterogeneous physical quantities onto a comparable scale, which facilitates their subsequent weighted fusion.

It should be noted that Equation (10) models the blur induced by rotational motion, which produces a globally consistent, depth-independent pixel displacement proportional to the focal length. Translational motion induces depth-dependent blur whose magnitude scales with $fvt_{\exp}/Z$ for scene points at depth *Z*; since per-pixel depth is unavailable before tracking is established, it cannot be evaluated frame-wise in the front end and is only partially covered by $R_{\exp}$ and $R_{\mathrm{motion}}$ as a conservative proxy. Likewise, the temporal alignment model of Equation (1) assumes a global-shutter sensor; rolling-shutter cameras expose different rows at different times, so the exposure-midpoint reconstruction would need to be generalized to row-wise timestamps. These extensions are discussed as limitations in Section 5.

Based on the above risk components, the joint observation risk score of the *k*-th image frame is defined as

(12) $$R_{k}=w_{1}R_{\exp}^{k}+w_{2}R_{\mathrm{gain}}^{k}+w_{3}R_{\mathrm{change}}^{k}+w_{4}R_{\mathrm{motion}}^{k},$$

where $w_{1}$, $w_{2}$, $w_{3}$, and $w_{4}$ are nonnegative weights governing the relative contributions of exposure duration, analog gain, inter-frame exposure–gain variation, and motion-blur risk to the overall joint risk evaluation. In practice, these weights can be set empirically or calibrated based on the camera’s imaging characteristics, operating environment, and front-end tracking performance. A higher $R_{k}$ value indicates that the current frame is subject to prolonged exposure, high analog gain, pronounced exposure–gain fluctuations, or severe motion blur, which corresponds to lower reliability of the associated visual observations. Building on this joint risk score, the image reliability metric for the current frame is further defined as

(13) $$q_{k}=\exp\left(-R_{k}\right),$$

where $q_{k}\in \left(0,1\right]$. As the joint risk score $R_{k}$ increases, $q_{k}$ decreases exponentially, indicating a reduction in the reliability of the current visual observation. Conversely, when $R_{k}$ remains small, $q_{k}$ approaches 1, suggesting that the current frame is more suitable for stable visual tracking. The risk score and image reliability metric are not used to directly discard image regions. Instead, they serve as adaptive control signals for feature extraction, KLT optical flow tracking, and outlier feature correspondence rejection.

#### 3.4.2. Adaptive Front-End Design

During the feature extraction stage, the quality level threshold of the GFTT corner detector is adaptively adjusted as

(14) $$Q_{\mathrm{GFTT}}=\mathrm{clip}\left(0.01+0.01\left(R_{\mathrm{gain}}^{k}+R_{\mathrm{change}}^{k}+R_{\mathrm{motion}}^{k}\right),\;0.01,\;0.04\right),$$

where $Q_{\mathrm{GFTT}}$ denotes the quality-level threshold of the Shi–Tomasi corner detector. $R_{\mathrm{gain}}^{k}$, $R_{\mathrm{change}}^{k}$, and $R_{\mathrm{motion}}^{k}$ represent the gain-related risk, inter-frame exposure–gain variation risk, and motion-blur risk, respectively. When the image is affected by high analog gain, pronounced exposure–gain variation, or severe motion blur, the proposed method automatically increases the corner detection threshold, enabling the front-end pipeline to preferentially retain features with stronger responses and more stable local structures. The exposure-time risk is not explicitly incorporated into this threshold, as the impact of prolonged exposure on feature quality also depends on the actual camera motion within the exposure interval, which is already captured by $R_{\mathrm{motion}}^{k}$.

During the KLT optical flow tracking stage, the optical flow parameters are adaptively tuned according to the motion-blur risk and inter-frame angular motion. First, the KLT search window size is adjusted based on the pixel-level motion-blur risk $B_{k}$ over the exposure interval as

(15) $$W_{LK}=\left\{\begin{array}{cc} 21, & B_{k}\leq 2\\ 25, & 2<B_{k}\leq 4\\ 31, & B_{k}>4 \end{array}\right.,$$

where $W_{\mathrm{LK}}$ denotes the local search window size of the KLT tracker. When $B_{k}$ is small, the local image structure remains relatively sharp, and a small search window is sufficient for stable tracking. As $B_{k}$ increases, motion blur smears the local intensity distribution, and enlarging the search window improves the robustness of optical flow estimation. Furthermore, the number of image pyramid levels is adaptively tuned according to the angular motion between the exposure midpoints of two consecutive frames. The inter-frame angular displacement is defined as

(16) $$\theta_{\mathrm{frame}}^{k}=\int_{t_{\mathrm{mid}}^{k-1}}^{t_{\mathrm{mid}}^{k}}∥\omega \left(t\right)∥dt,$$

where $t_{\mathrm{mid}}^{k-1}$ and $t_{\mathrm{mid}}^{k}$ denote the exposure-midpoint timestamps of the (k−1)-th and *k*-th image frames, respectively. The number of pyramid levels in the KLT tracker is then determined as

(17) $$L_{pyr}=\left\{\begin{array}{cc} 3, & \theta_{\mathrm{frame}}\leq \tau_{\theta }\\ 4, & \theta_{\mathrm{frame}}>\tau_{\theta } \end{array}\right.,$$

where $L_{\mathrm{pyr}}$ denotes the number of pyramid levels employed in the KLT tracker, and $\tau_{\theta }$ corresponds to the threshold for inter-frame angular motion. When pronounced angular motion occurs between consecutive frames, increasing the number of pyramid levels expands the coarse-to-fine search range, thus improving the tracking success rate under large displacements. Accordingly, the forward–backward consistency threshold is adaptively adjusted. The reliability of a feature correspondence is evaluated by comparing the feature’s original position in the previous frame with the position obtained by first tracking it forward to the current frame and then backward to the previous frame. The forward–backward consistency threshold $\tau_{\mathrm{fb}}$ is defined as

(18) $$\tau_{\mathrm{fb}}=\mathrm{clip}\left(0.5+0.3R_{\mathrm{motion}}^{k},\;0.5,\;1.2\right)$$

Motion blur amplifies the uncertainty of optical flow estimation. Accordingly, the forward–backward consistency threshold is moderately relaxed as motion-blur risk increases, thereby preserving potentially valid feature tracks under blurred imaging conditions. In contrast, the threshold is not relaxed in response to high sensor gain or inter-frame exposure–gain variations, as these factors primarily degrade local brightness consistency. Relaxing the forward–backward consistency constraint under such circumstances is more likely to introduce erroneous correspondences. After KLT tracking, patch-wise photometric consistency filtering is further applied to each matched feature. For the *i*-th feature tracked via KLT, the corresponding local image patches are extracted from the (k−1)-th and *k*-th frames as

(19) $$P_{i}^{k-1},\;P_{i}^{k},$$

where $P_{i}^{k-1}$ and $P_{i}^{k}$ denote the local image patches centered at the corresponding feature in the (k−1)-th and *k*-th image frames, respectively. The ratio of saturated pixels within the current patch is first computed as

(20) $$s_{i}^{k}=\frac{1}{\left|P_{i}^{k}\right|}\sum_{x\in P_{i}^{k}}I\left(I_{k}\left(x\right)>T_{\mathrm{sat}}\right),$$

where $\left|P_{i}^{k}\right|$ denotes the total number of pixels in the patch, I(·) is the indicator function, and $T_{\mathrm{sat}}$ is the saturation threshold. For 8-bit grayscale images, $T_{\mathrm{sat}}$ is set to 245 to identify locally overexposed regions. If the saturation ratio of either patch in two consecutive frames satisfies

(21) $$s_{i}^{k-1}>\tau_{s}\;\mathrm{or}\;s_{i}^{k}>\tau_{s}$$

the local region associated with the feature is considered severely overexposed, and its intensity structure is regarded as unreliable. The corresponding feature is therefore discarded. For the features that pass the saturation ratio test, the ZNCC between the corresponding local patches in the two consecutive frames is further computed as

(22) $${\mathrm{ZNCC}}_{i}^{k}=\frac{\sum_{x}\left(P_{i}^{k-1}\left(x\right)-{\bar{P}}_{i}^{k-1}\right)\left(P_{i}^{k}\left(x\right)-{\bar{P}}_{i}^{k}\right)}{\sqrt{\sum_{x}{\left(P_{i}^{k-1}\left(x\right)-{\bar{P}}_{i}^{k-1}\right)}^{2}}\sqrt{\sum_{x}{\left(P_{i}^{k}\left(x\right)-{\bar{P}}_{i}^{k}\right)}^{2}}},$$

where ${\bar{P}}_{i}^{k-1}$ and ${\bar{P}}_{i}^{k}$ denote the mean intensity values of the corresponding patches in the (k−1)-th and *k*-th image frames, respectively. By removing the mean intensity, ZNCC reduces the influence of global brightness shifts and primarily measures the consistency of local texture structures. If ${\mathrm{ZNCC}}_{i}^{k}$ satisfies

(23) $${\mathrm{ZNCC}}_{i}^{k}<\tau_{\mathrm{zncc}}$$

the local intensity structure of the corresponding patches is considered to have changed significantly. Therefore, even if KLT reports a successful track, the feature does not satisfy the local brightness consistency assumption and is discarded. In addition, the LK photometric residual $e_{i}^{k}$ obtained during KLT tracking is used for a second-stage consistency check. If

(24) $$e_{i}^{k}>\tau_{\mathrm{LK}},$$

the local matching error of the feature is considered excessively large, and the correspondence is discarded. The LK photometric residual threshold is adaptively adjusted according to the image reliability of the current frame, $q_{k}$, as

(25) $$\tau_{\mathrm{LK}}=\mathrm{clip}\left(30q_{k},\;8,\;30\right),$$

where clip(·) denotes the clipping function that constrains the threshold within a predefined range. When the current frame has a low observation risk, $q_{k}$ approaches 1 and $\tau_{\mathrm{LK}}$ approaches 30, allowing the level of optical flow residuals expected under normal imaging conditions. As the image reliability decreases because of high gain, significant exposure–gain variation, or severe motion blur, $q_{k}$ decreases accordingly, leading to a smaller $\tau_{\mathrm{LK}}$. This results in a stricter rejection criterion for features with large photometric residuals. Consequently, a hierarchical patch filtering strategy is established, consisting of saturation ratio filtering, ZNCC-based structural consistency verification, and adaptive LK residual filtering.

Finally, when the inter-frame angular motion is significant and the exposure-induced motion-blur risk is high, the feature locations in the current frame are predicted using the IMU rotation integrated over the interval, and the predicted positions are used as the initial estimates for KLT optical flow tracking. The IMU rotation increment between two consecutive frames is expressed as

(26) $$\Delta R_{I}^{k-1,k}=\prod_{t_{k-1}}^{t_{k}}\mathrm{Exp}\left(\omega \left(t\right)\Delta t\right),$$

where $\Delta R_{I}^{k-1,k}$ denotes the rotation increment of the IMU from the (k−1)-th frame to the *k*-th frame in the IMU coordinate system, Exp(·) is the exponential map on the Lie group SO(3), and Δt is the time interval between two consecutive IMU measurements. In practice, the above integration is approximated using the discrete sequence of IMU angular velocity measurements. Since visual features are defined in the camera coordinate system, the rotation increment must be transformed from the IMU coordinate system to the camera coordinate system as

(27) $$\Delta R_{C}^{k-1,k}=R_{CI}\cdot \Delta R_{I}^{k-1,k}\cdot R_{IC},$$

where $R_{CI}$ denotes the extrinsic rotation matrix from the IMU coordinate system to the camera coordinate system, and $R_{IC}$ denotes its inverse transformation. The feature locations in the previous frame are then propagated using the predicted camera rotation as

(28) $${\hat{p}}_{i}^{k}=\pi \left(\Delta R_{C}^{k-1,k}\cdot \pi^{-1}\left(p_{i}^{k-1}\right)\right),$$

where $p_{i}^{k-1}$ denotes the feature location in the (k−1)-th frame, ${\hat{p}}_{i}^{k}$ represents its predicted location in the *k*-th frame, π(·) refers to the pinhole camera projection function, and $\pi^{-1}\left(\cdot \right)$ denotes the back-projection function. This prediction depends exclusively on the estimated rotation and excludes translational motion components. Inter-frame rotation generally induces significant and globally consistent pixel displacements on the image plane, while displacements arising from translation are highly dependent on scene depth. Without reliable depth prior information, the introduction of translational prediction may yield inaccurate initial estimates owing to depth uncertainty. Accordingly, the proposed method leverages IMU rotation integration to provide initial feature positions for KLT tracking, and the subsequent optical flow optimization compensates for local displacements caused by translational motion.

#### 3.4.3. Summary

In summary, the proposed exposure–gain–motion-aware front-end enhancement fully exploits the exposure and gain information delivered by industrial cameras, as well as the motion information acquired from the hardware-synchronized IMU. The presented strategy enhances the VINS-Fusion front end via a complete pipeline covering imaging and motion state acquisition, inter-frame risk evaluation, adaptive feature extraction and KLT tracking, patch-level consistency filtering, and IMU-assisted rotation prediction. Under challenging scenarios involving complex illumination variations, automatic exposure adjustment, high-gain low-light imaging, and fast motion, the proposed method effectively suppresses unstable feature points and spurious correspondences, improves front-end tracking performance, and yields more reliable feature observations for back end visual–inertial optimization.

## 4. Experiments and Evaluation

**Figure 6:**
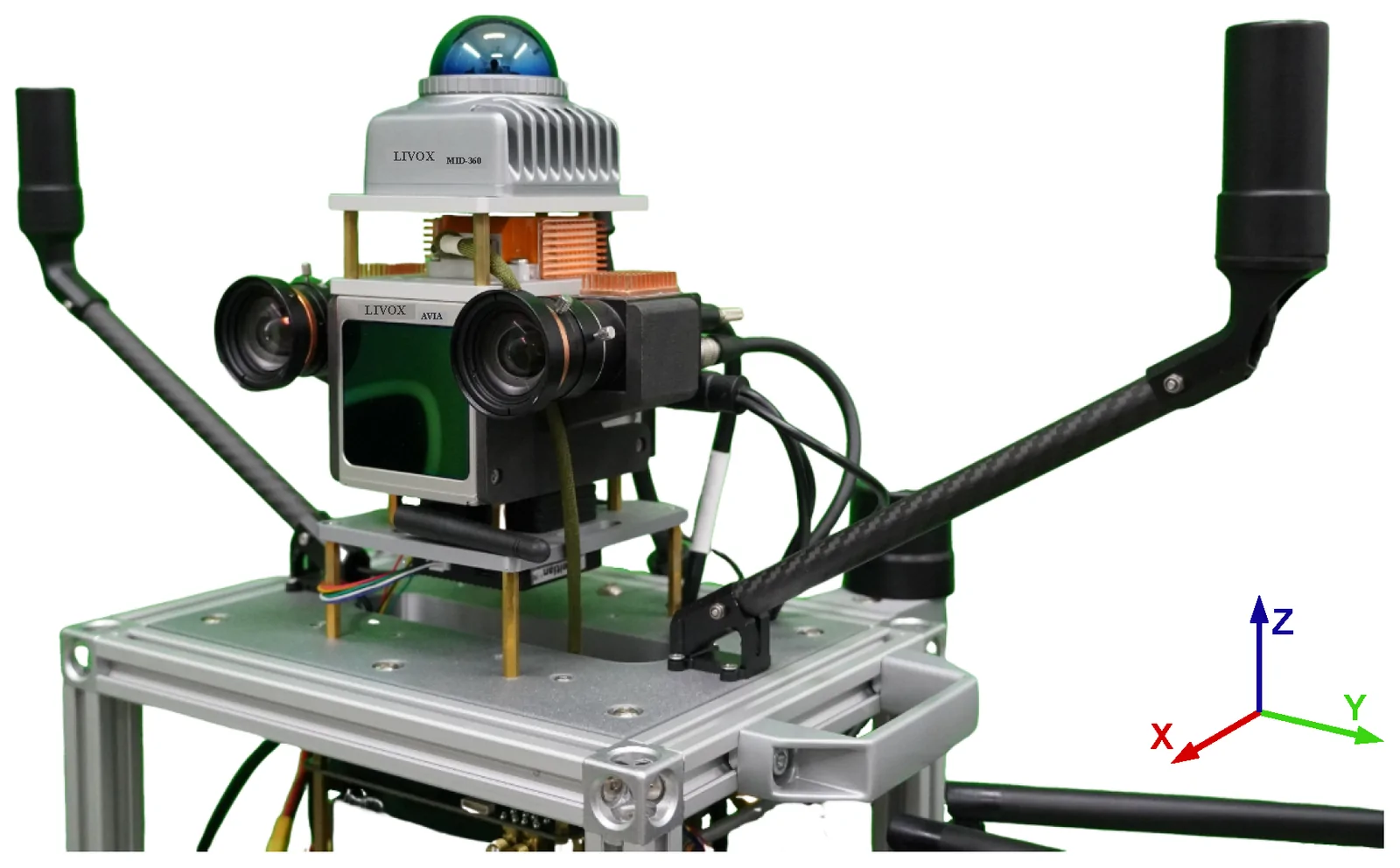
Experimental platform for exposure-midpoint temporal alignment.

As illustrated in Figure 6, the experimental platform consists of a STIM300 inertial measurement unit (IMU; Safran Sensing Technologies Norway AS, Horten, Norway), two forward-looking global-shutter RGB cameras (MV-CA013-20GC; HIKROBOT, Hangzhou, China), two solid-state LiDARs (Livox Mid-360 and Avia; Livox Technology, Shenzhen, China), and a dual-antenna GNSS RTK receiver (BG-410; Shenzhen Beitian Communication Co., Ltd., Shenzhen, China), all rigidly mounted and synchronized by the Multi-Sensor Hardware Time Synchronization System (MS-HTSS) described in Section 3.3. The STIM300 serves as the origin and reference of the platform coordinate system and provides the three-axis gyroscope and accelerometer measurements used by the VIO system. The two RGB cameras are triggered synchronously at 25 Hz by MS-HTSS and operate in auto-exposure and auto-gain modes; each trigger event is hardware timestamped, and the frame-wise exposure duration and analog gain are recorded together with the image, providing the metadata required by the temporal alignment model presented in Section 3.3 and the risk model presented in Section 3.4.1. The stereo cameras and the STIM300 are the only sensors involved in state estimation. The Livox Mid-360, together with the hardware-synchronized IMU, is used to generate reference trajectories with Faster-LIO for the indoor experiments, and the dual-antenna RTK receiver provides a centimeter-level absolute position reference for the outdoor experiments; both serve exclusively as ground truth sources and are not used for estimation.

### 4.1. Temporal Alignment Experiments

**Figure 7:**
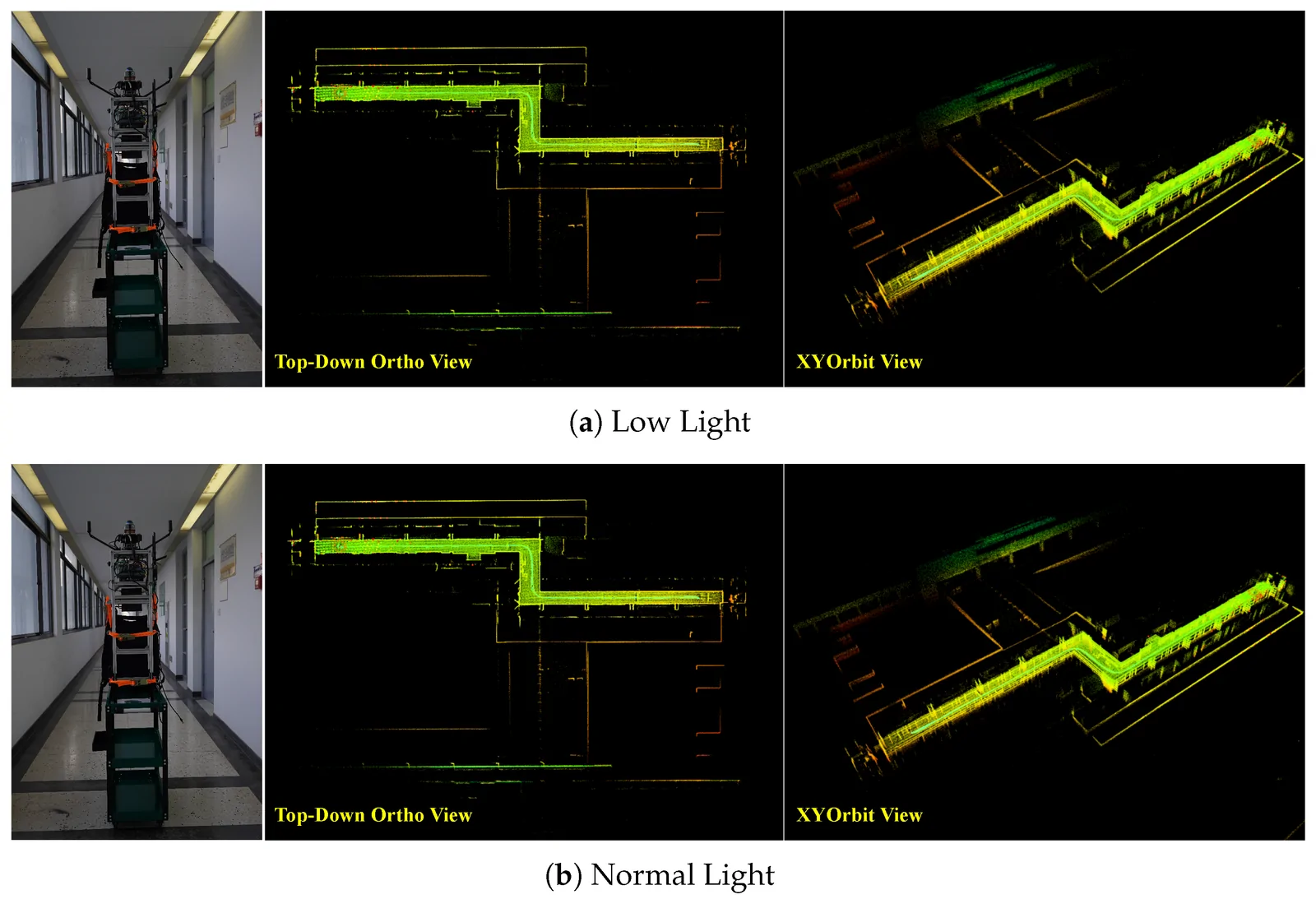
Point cloud maps of indoor scenes reconstructed using Faster-LIO under different illumination conditions. Points are colored by height (elevation) relative to the map origin.

**Figure 8:**
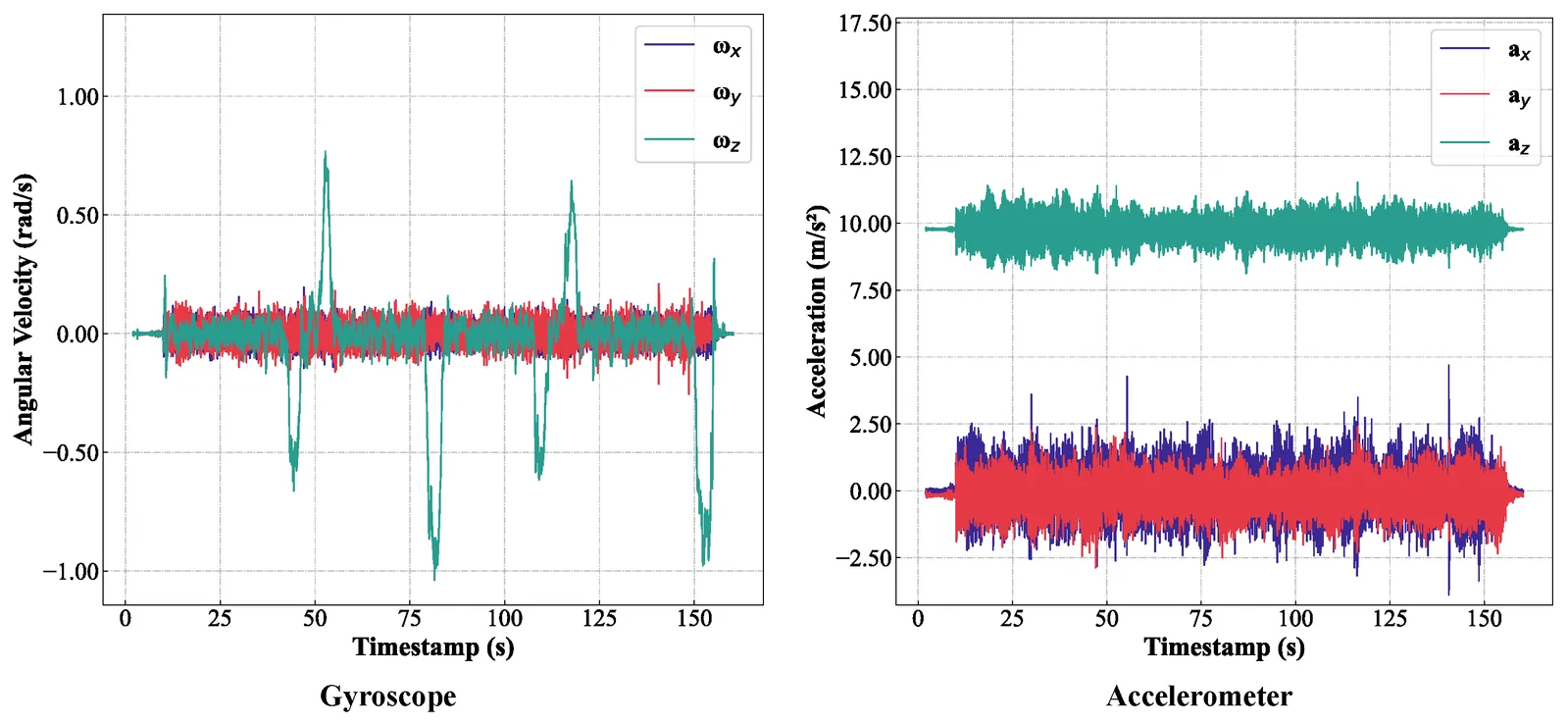
Raw IMU data waveforms in low-light indoor corridor scenario.

**Figure 9:**
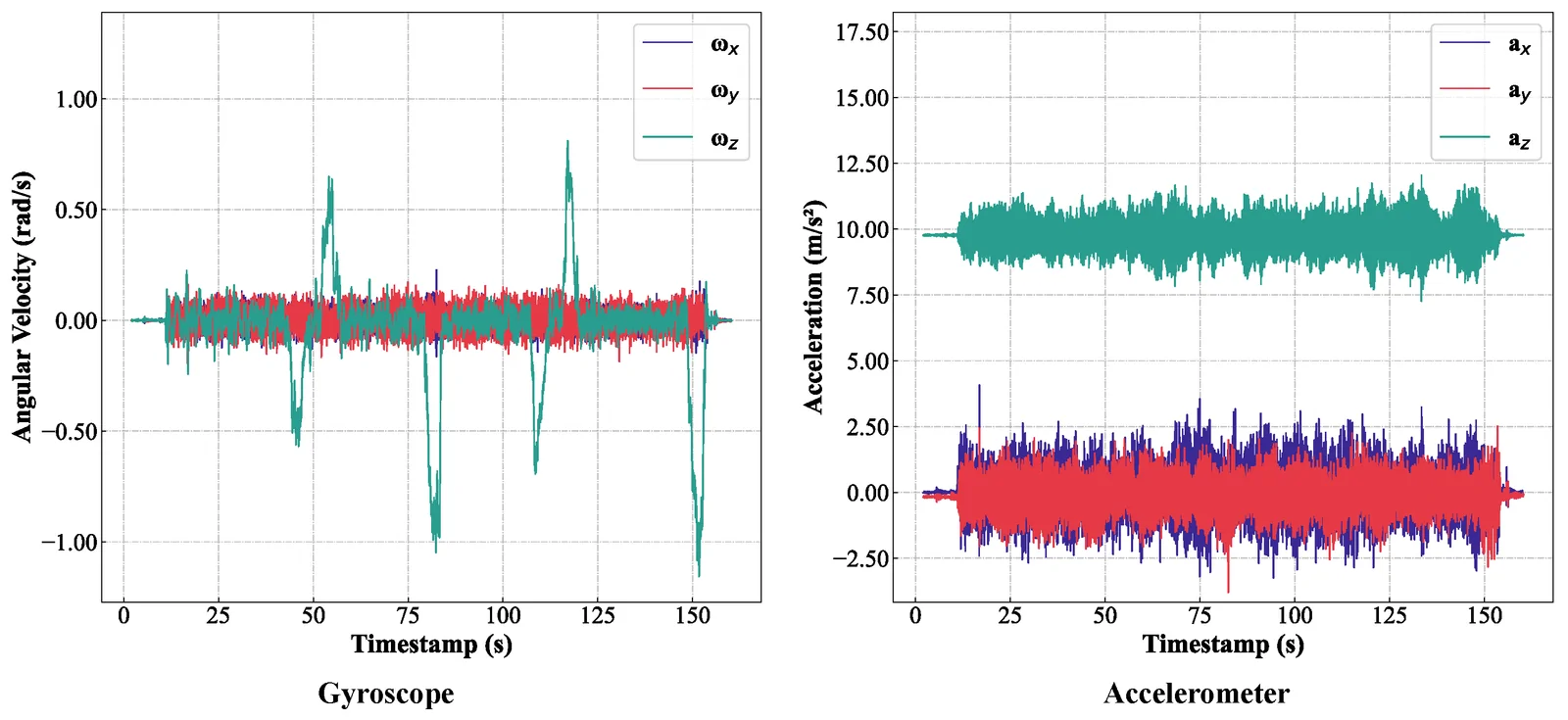
Raw IMU data waveforms in normal-light indoor corridor scenario.

To quantitatively evaluate the impact of the temporal alignment scheme described in Section 3.3 on the accuracy of the VINS algorithm, this experiment uses a customized cart-mounted acquisition platform to collect two sequences under different illumination conditions in the same indoor corridor. The Faster-LIO algorithm [43] is employed to generate a reference trajectory based on the hardware-synchronized IMU and Livox Mid-360 LiDAR measurements, which is used for subsequent evaluation of the accuracy of the VINS algorithm. The reconstructed point cloud map of the test scene is shown in Figure 7. The start and end positions of the two data sequences are exactly identical. Their raw IMU waveforms are shown in Figure 8 and Figure 9. It can be observed that the two sequences exhibit similar smooth motion states and nearly identical durations.

#### 4.1.1. Dual Delay–Source Decomposition and Controlled Experiment Design

As described in Section 3.3, Equations (2) and (3) indicate that the camera–IMU temporal error entering the back-end estimator consists of two physical sources: the constant output delay $t_{\mathrm{Total}}$ on the IMU side and the exposure-midpoint offset $\Delta t_{\mathrm{IC}}^{k}$ on the image side, which varies with exposure time across frames. These two components have fundamentally different characteristics. The former is constant and independent of exposure, making it exactly the type of offset that fixed or online offset estimators can effectively absorb. The latter varies from frame to frame and is strongly correlated with exposure time, which cannot be approximated by any constant scalar. To quantitatively analyze their effects on the trajectory accuracy of the VINS algorithm, a controlled experiment is designed in which the image timestamp is the only variable while all other factors remain unchanged using the same dataset.

**Figure 10:**
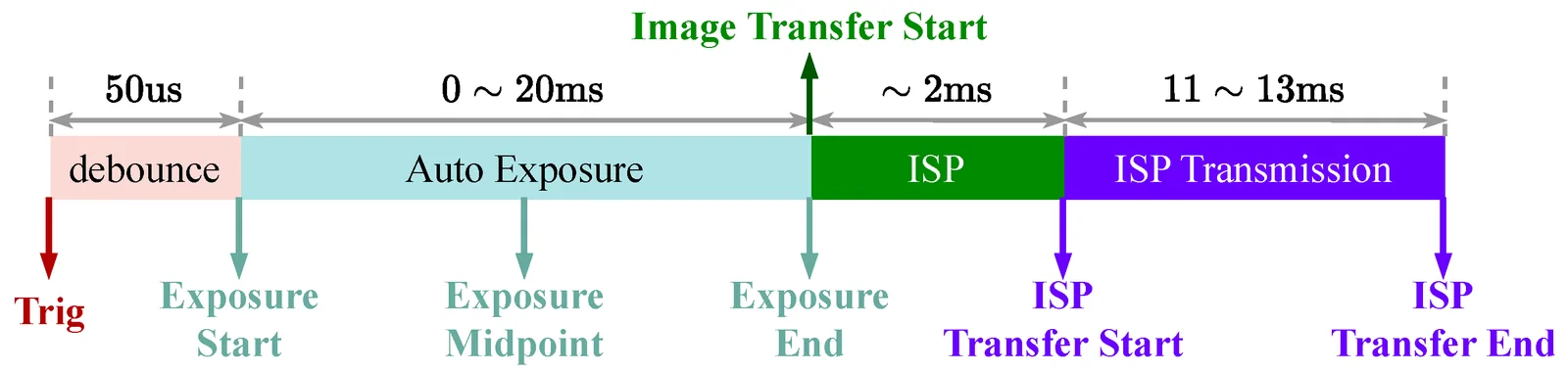
Timing of industrial camera data collected in trigger mode.

Figure 10 shows the timing of the industrial camera operating in trigger mode. By reconstructing the exposure-midpoint timestamp frame by frame on the shared hardware clock (Equation (1)), MS-HTSS removes the exposure-dependent component from the frame timestamps and, at the same time, stamps them without the host reception and ROS dispatch delays inherent to software-based acquisition.

**Figure 11:**
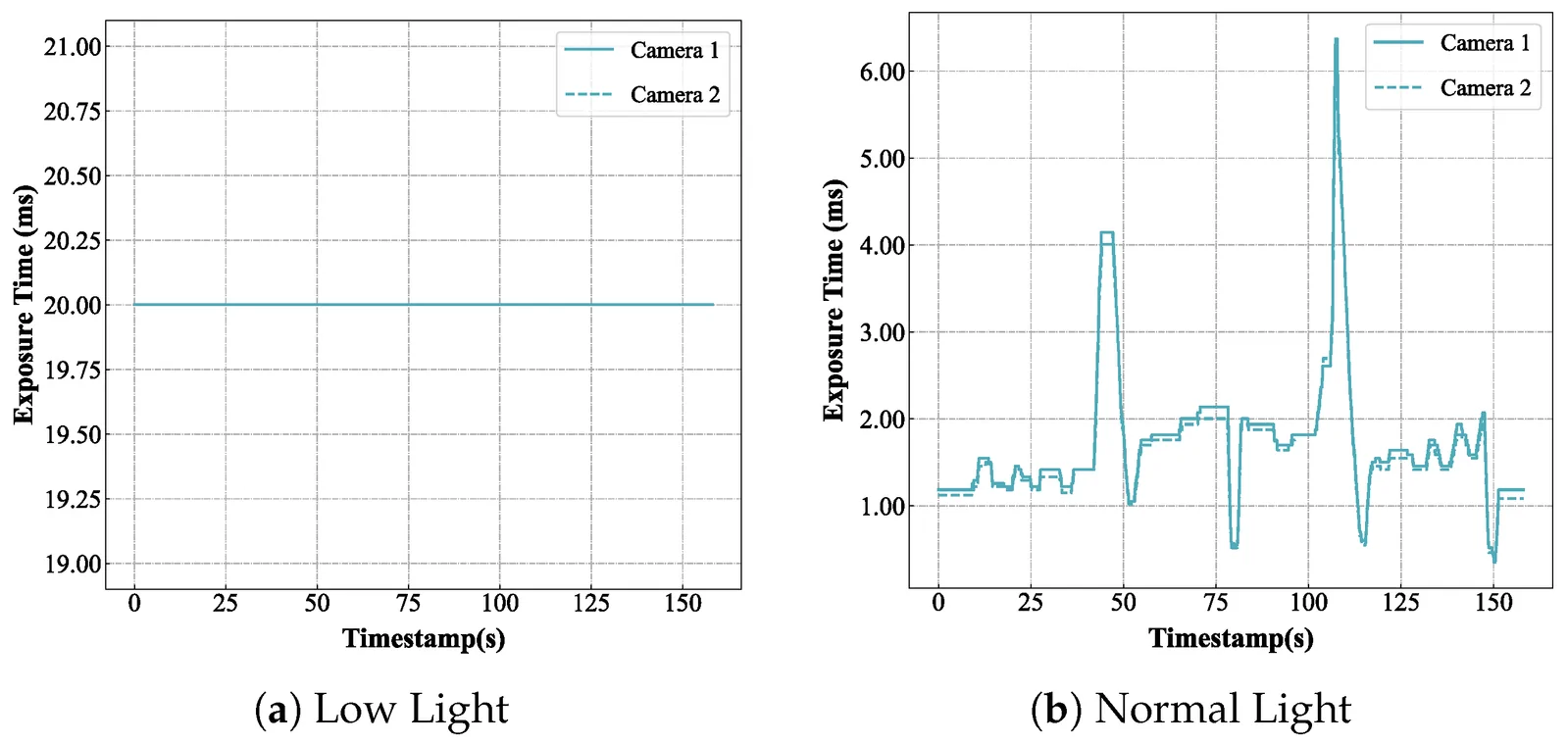
Exposure time of industrial cameras under different lighting conditions.

Based on this setup, the controlled experiment uses the exposure-midpoint timestamp reconstructed using MS-HTSS as the reference timestamp for the two illumination sequences. The exposure-start and exposure-end test timestamps are reconstructed offline from the same ROS 2 bag file by shifting the image timestamps before and after the exposure midpoint by half of the exposure time. The exposure curve in Figure 11 provides the key basis for this experimental design. Under low-light conditions, AE sets the exposure time to a constant value of 20ms, resulting in exact offsets of −10ms and +10ms for the exposure-start and exposure-end timestamps relative to the midpoint, respectively. Under normal illumination conditions, AE adjusts the exposure time frame by frame between 0.4 and 6.4ms, corresponding to frame-dependent offsets of ±(0.2–3.2)ms. The offset compensation strategy is treated as the second experimental factor. The fixed-offset strategy uses a single offset of 13.828ms obtained using Kalibr calibration, while the online strategy obtains the time offset through online estimation using VINS-Fusion.

#### 4.1.2. Comparative Experimental Results of Controlled Timestamps

**Figure 12:**
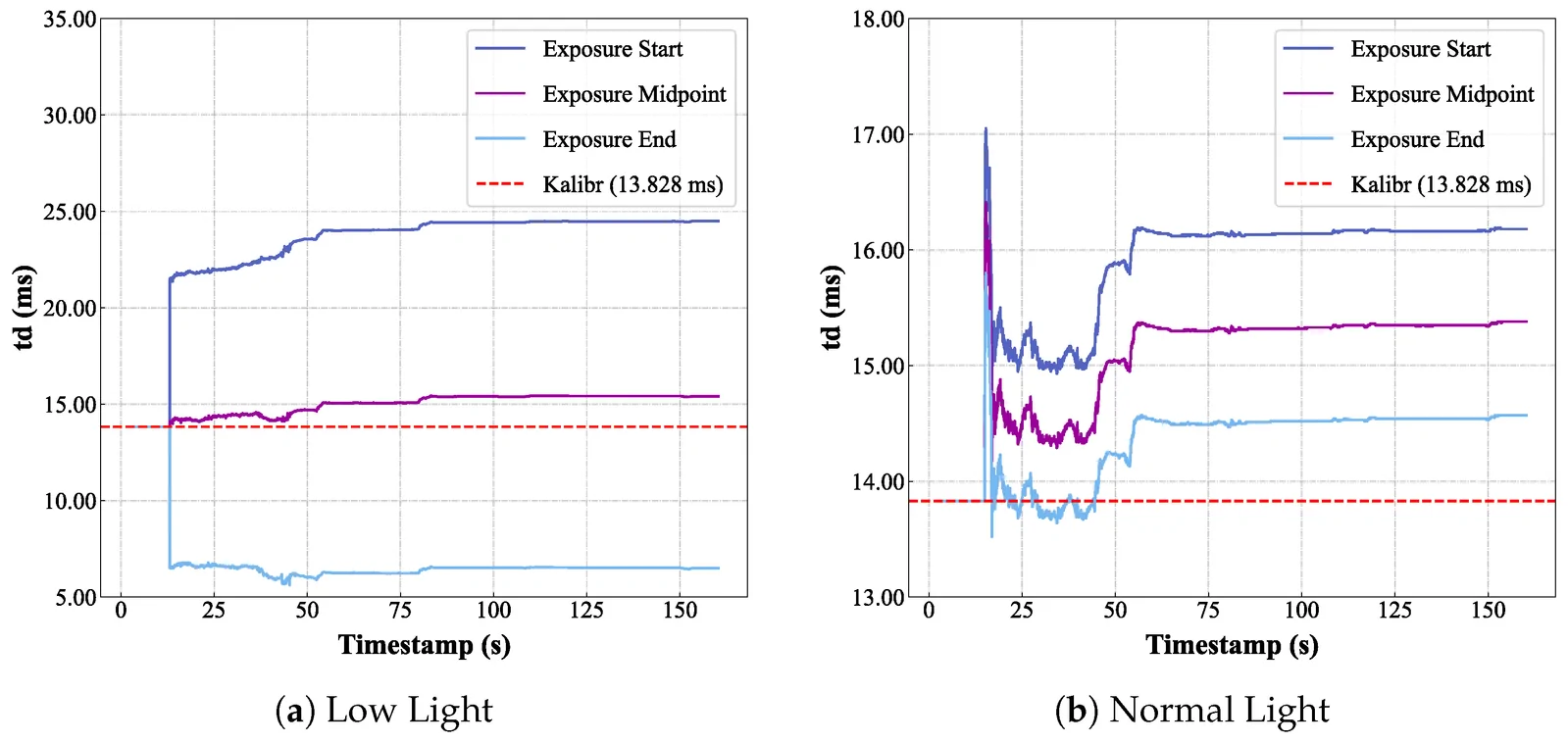
Online time offset estimation curve using VINS-Fusion.

The EVO toolkit is used to evaluate the RMSE and STD of ATE and RPE, and the quantitative results are presented in Table 2 and Table 3. The time offset estimated online using VINS-Fusion is shown in Figure 12.

Table 2 and Table 3 provide the complete RMSE/STD statistics of ATE and RPE under the two scenarios, grouped by timestamp reference type and offset compensation strategy. To intuitively analyze the individual effects of the frame timestamp reference and offset compensation strategy on the VINS trajectory accuracy, and to highlight the orthogonal structure between these two variables, the ATE RMSE values that directly support the analysis are extracted from Table 2 and Table 3 to form the compact table shown in Table 4.

#### 4.1.3. Result Analysis and Cross-Validation

The controlled experiment treats the frame timestamp reference and the offset compensation strategy as two orthogonal experimental factors. The influence of the timestamp reference becomes significant only when the exposure time is substantially extended. Under low-light conditions, AE sets the exposure time to 20ms, resulting in exact offsets of −10ms and +10ms for the exposure-start and exposure-end reference timestamps relative to the exposure midpoint, respectively. The midpoint reference achieves the lowest ATE RMSE under both fixed and online strategies, with values of 0.434m and 0.415m, respectively (Table 4). Compared with the exposure-start reference, the midpoint reference reduces the ATE RMSE by 28.6% for the fixed strategy and 22.7% for the online strategy. Compared with the exposure-end reference, the reductions are 12.9% and 19.2%, respectively. Under normal illumination conditions, the exposure time only varies between 0.4 and 6.4ms, and the offsets between the exposure-start/end reference timestamps and the exposure midpoint remain within ±(0.2–3.2)ms. The ATE RMSE values of the three timestamp references differ within ±5%. The comparison of the two illumination conditions demonstrates that the improvement obtained from the midpoint reference originates from removing the exposure-related frame-wise temporal component. This component only becomes sufficiently large enough to dominate trajectory accuracy when AE extends the exposure time to the order of 20ms. The ATE STD under the midpoint reference is also reduced by nearly half (Table 2). For the fixed strategy, it decreases from 0.373m to 0.198m, and for the online strategy, it decreases from 0.316m to 0.186m. Meanwhile, the RPE RMSE remains stable across all timestamp references and illumination conditions, ranging from 0.063 to 0.066m under low-light conditions and from 0.044 to 0.045m under normal illumination. These results indicate that the benefit of temporal alignment is mainly reflected in global trajectory consistency rather than local accuracy.

Under the unified timestamp reference, the fixed-offset and online estimation strategies achieve ATE RMSE values at the same level, with 0.434m and 0.415m under low-light conditions, and 0.402m and 0.393m under normal illumination conditions, respectively. The online strategy only reduces the error by approximately 2–4%. This equivalence is theoretically supported by the dual-source temporal error model. With the midpoint reference timestamp, the temporal error entering the estimator is reduced to a constant output delay $t_{\mathrm{Total}}$ that is independent of exposure. Both fixed compensation using the Kalibr-calibrated offset and online estimation can equivalently absorb this constant delay. The estimated time offsets further verify this interpretation. The mean time offsets estimated online under low-light and normal illumination conditions converge to 14.979ms and 15.045ms, respectively (Figure 12), with a difference of only 0.066ms. This is observed despite the exposure times of the two scenarios differing by approximately one order of magnitude. If the exposure-midpoint timestamp reconstruction was ineffective and the frame timestamps still represented the trigger instants, the estimated offsets of the two sequences would be expected to differ by approximately 7–9ms. The value and physical interpretation of the estimated time offset depend on the frame timestamp reference. Therefore, the baselines of fixed compensation, online estimation, and the ideal physical delay are only meaningful when compared under the same timestamp reference. This justifies the orthogonal design of the two factors in the matrix experiment and further indicates that fixed and online strategies are not competing approaches, but consistent solutions that can be selected according to implementation cost.

The online estimated time offset represents the operating point of the back-end estimator rather than an unbiased measurement of the physical delay. Under smooth indoor motion, the observability of the time offset is limited, and part of the temporal error is coupled with other states such as velocity and bias. Consequently, the online estimation responds sublinearly to shifts in the timestamp reference and retains an offset of approximately 3ms. If the estimation was unbiased, the low-light exposure-end, midpoint, and exposure-start references would correspond to approximately 3.8ms, 13.8ms, and 23.8ms, respectively. However, the measured mean values are 6.921ms, 14.979ms, and 23.152ms. Therefore, directly interpreting the online scalar estimate as the physical delay and comparing it numerically with the Kalibr result leads to apparent inconsistencies. Three independent sources of evidence provide mutual verification. The Kalibr calibration converges to 13.828ms, the online estimation converges to 14.979ms and 15.045ms under low-light and normal illumination conditions, respectively, and the total gyroscope channel delay reported in the STIM300 documentation is approximately 13.2ms. These values differ by only 0.6–1.9ms. Since the observability of the time offset is mainly provided by rotational motion, the estimated scalar offset is anchored near the gyroscope channel delay. This indicates that $t_{\mathrm{Total}}$ is a real constant delay rather than an artifact introduced by the estimation algorithm.

In summary, exposure-midpoint timestamp reconstruction does not eliminate all temporal errors. Instead, the residual errors are reduced to two identifiable components: the camera-side reporting residual $\delta t_{\mathrm{cam}}$, which includes the discrepancy between the reported exposure value and the actual integration window as well as the instantaneous deviation introduced by AE adjustment, and the approximately 4.3ms documented delay difference between the gyroscope and accelerometer channels. No single scalar offset can simultaneously compensate for both channels. Both components are constant terms at the sub-millisecond to millisecond level and can be absorbed by the estimator. Therefore, the applicability boundary of the proposed method becomes clear. The benefit of hardware-level exposure-midpoint reconstruction mainly appears in long-exposure and highly dynamic exposure scenarios, where AE significantly extends the exposure time. For normal short-exposure scenarios, it achieves an equivalent performance to fixed-offset compensation without introducing additional cost.

### 4.2. Ablation Study of the Adaptive Front End

The adaptive front end consists of several modules that can be independently enabled or disabled. To facilitate the description and support module-wise ablation studies, Table 5 summarizes the module identifiers, functions, and corresponding roles. The identifiers are used throughout the following sections for concise reference.

This section evaluates the impact of each enhancement module in Table 5 on trajectory accuracy through module-wise ablation experiments on two sequences collected by the self-developed experimental platform. The descriptions of the experimental sequences are provided in Section 4.2.1, while the baseline definition and configuration settings of the ablation study are described in Section 4.2.2.

#### 4.2.1. Description of Experimental Sequences

This section describes the sequences used for the subsequent evaluation. Sequence 1 is collected in a multi-floor indoor corridor, while Sequence 2 is collected on roads between campus buildings. Table 6 lists the acquisition method, scenario, ground truth source, and motion statistics for each sequence.

The roles of the two sequences are complementary. Sequence 1 contains challenging low-illumination segments, and the configuration of its environment differs from the controlled sequence in Section 4.1, featuring a multi-floor structure and closed corridors. It is used to verify whether the conclusions remain valid under more complex geometric configurations and more substantial imaging challenges. Sequence 2 is collected during outdoor cycling under strong sunlight and high-contrast imaging conditions, with a peak velocity of approximately 10m/s. Its linear velocity and vibration level are significantly higher than those of walking sequences, making it suitable for evaluating the performance of the proposed method under outdoor imaging conditions and more intensive motion dynamics.

**Figure 13:**
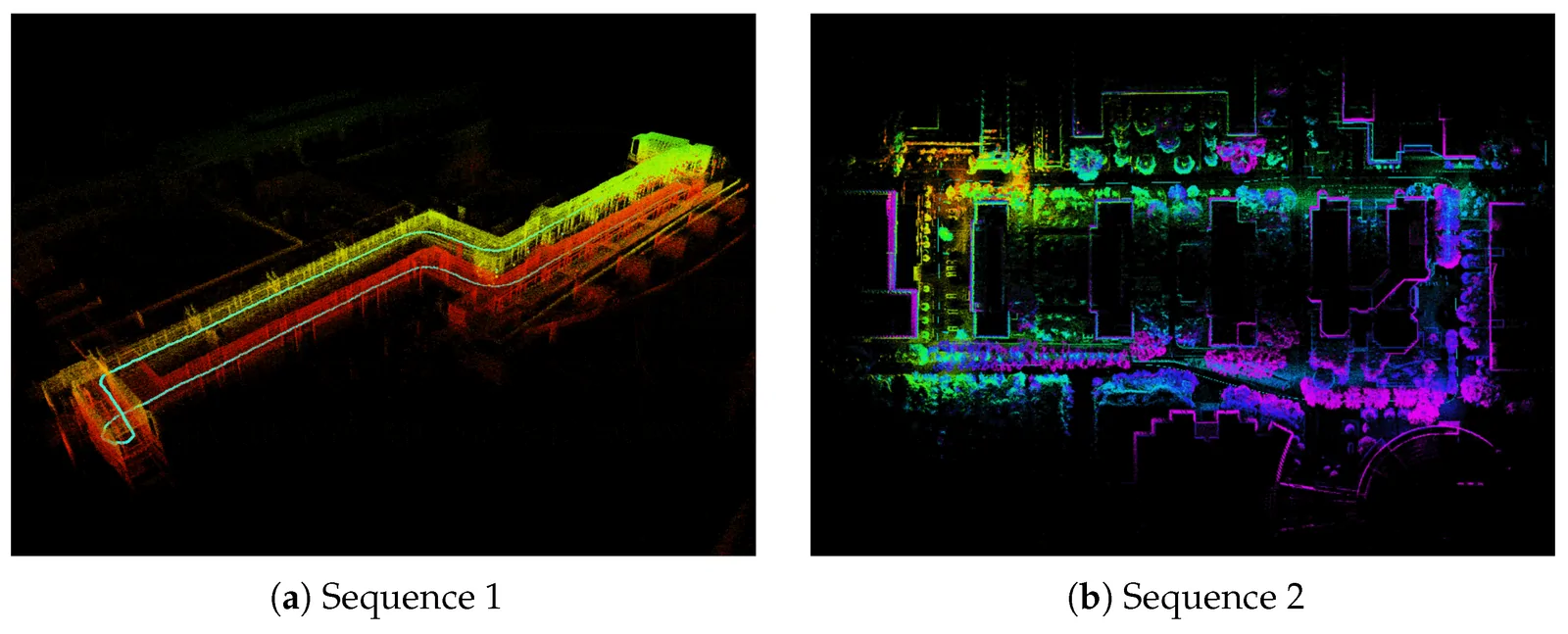
Point cloud maps of the two sequences reconstructed using Faster-LIO. Points are colored by height (elevation) relative to the map origin.

**Figure 14:**
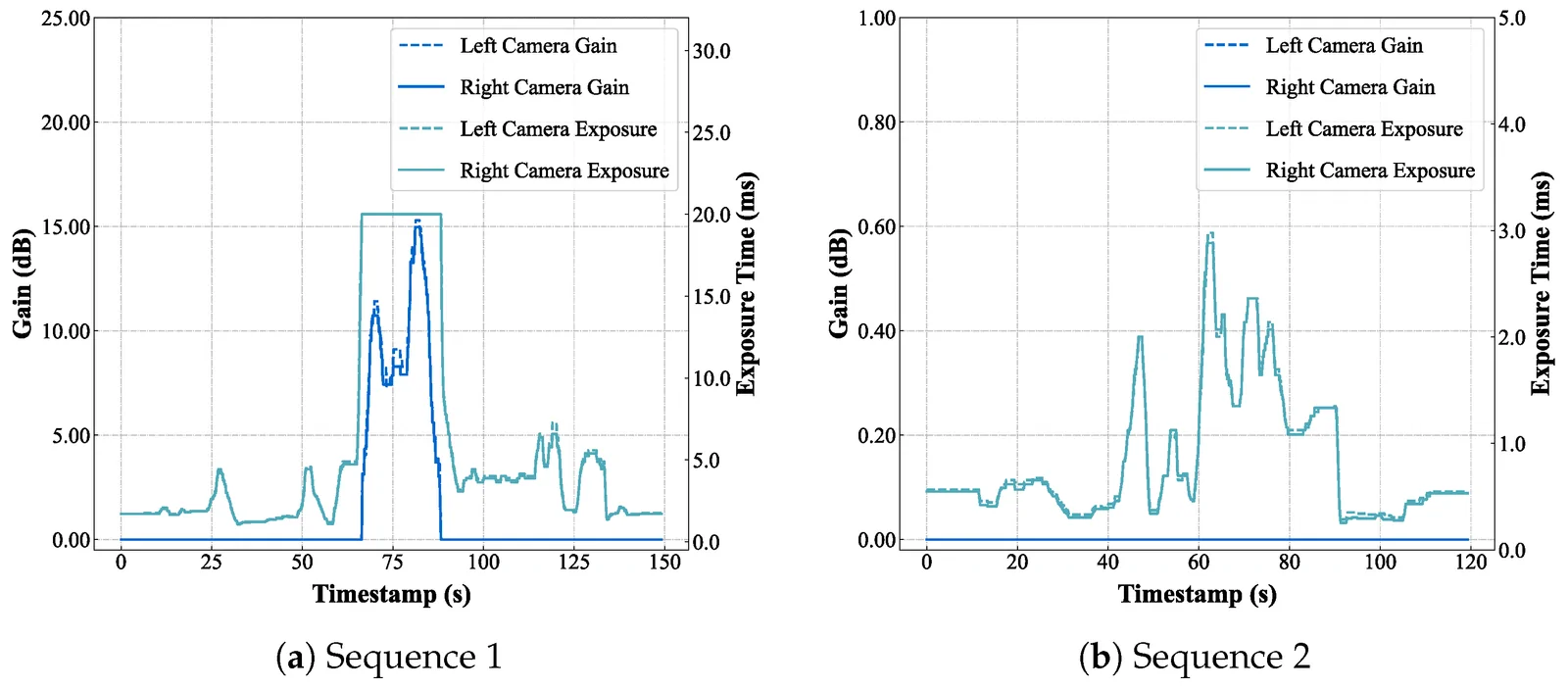
Exposure time and gain of industrial cameras under different scenarios.

Figure 13 shows the scene maps of the two sequences, and Figure 14 presents the exposure and gain profiles of Sequence 1 and Sequence 2.

#### 4.2.2. Per-Module Ablation Results

**Figure 15:**
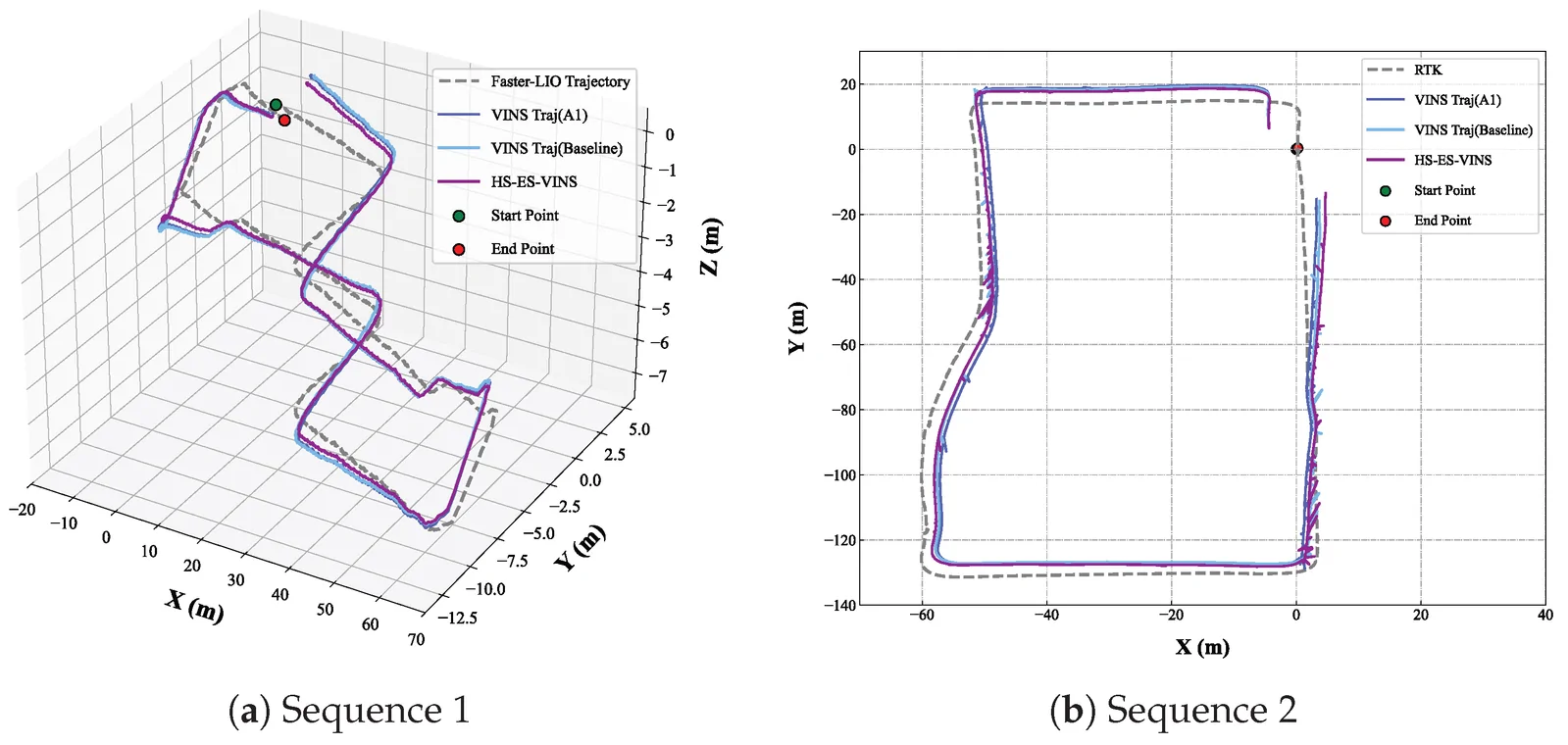
Trajectory comparison of the baseline VINS-Fusion, exposure-midpoint temporal alignment, and HS-ES-VINS under different scenarios.

The ablation study follows a progressive accumulation strategy starting from the baseline. The baseline is the original VINS front end with Kalibr-calibrated fixed offset compensation, where the image timestamp corresponds to the exposure-end time. This setting is consistent with the timestamp convention used for the Kalibr baseline rows in Section 4.1. The fixed offset is identical for all configurations and is not recalibrated for different sequences. The semantics of each configuration are as follows. A1 denotes exposure-midpoint timestamp alignment. A1 + A3 applies the fixed threshold mechanism on top of A1. A1 + A3 + A4 enables the A4 module driven by the frame-wise risk estimated by A2, where the LK residual threshold in A3 is also adaptively adjusted according to A2. Finally, A1 + A3 + A4 + A5 represents the complete front end. Each sequence is evaluated independently to isolate the influence of sequence variations on the contribution of each module. The corresponding results are presented in Table 7 and Table 8. The trajectory comparison results are shown in Figure 15.

Under the complete front-end configuration with all modules A1–A5 enabled, two operating modes are compared: the EGM risk evaluation mode with A2 enabled and the mode with A2 disabled. When A2 is disabled, all risk components are fixed to constant values, causing the adaptive thresholds in A3, A4, and A5 to degenerate into fixed thresholds. The evaluation results for the two sequences are presented in Table 9.

The complete front end reduces the ATE RMSE of the two sequences by 16.8% and 12.9% compared with the baseline, while the ATE STD decreases by 21.2% and 12.8%, respectively. Moreover, all configurations show monotonic improvement along the module accumulation process. The effect on RPE is not consistent across the two sequences, as different modules influence local motion consistency in different directions. The RPE RMSE increases by 2.6% and 1.7% compared with the baseline, respectively. Therefore, the benefit of the proposed front end is mainly reflected in global trajectory accuracy.

Analyzing the contribution of individual modules, the effect of A1 is consistent with the controlled experiment in Section 4.1. For Sequence 2, where the exposure time remains at only a few milliseconds, A1 changes the ATE RMSE by only 0.6%, which is within the noise level, while the ATE STD and RPE STD are improved significantly. Sequence 1 contains approximately 20s of low-light segments where the exposure time is set to 20ms. In this case, A1 reduces the ATE RMSE and RPE RMSE by 1.7% and 9.8%, respectively, confirming that the benefit of temporal alignment originates from the exposure-related frame-wise component under long-exposure conditions. A3 provides the largest contribution to ATE improvement. Starting from A1, A3 further reduces the ATE RMSE of the two sequences by 9.3% and 7.0%, respectively. This improvement is observed under both indoor corridor and outdoor high-contrast imaging conditions. A4 and A5 provide additional but smaller improvements, reducing the ATE RMSE by another 2.0% and 2.9%, and by 4.7% and 2.9%, respectively.

The isolation study in Table 9 further demonstrates the effectiveness of A2. When the front-end structure and adaptive threshold formulation remain unchanged, but all risk components provided by A2 are fixed to constant values, the ATE RMSE of the two sequences increases by 12.5% and 6.6%, respectively, while the ATE STD increases by 15.0% and 9.6%. These results indicate that the frame-wise risk information provided by A2 contributes directly to adaptive decision making rather than merely reproducing the effect of fixed-threshold adjustment.

#### 4.2.3. Feature Tracking Reliability Under Different EGM Risk Levels

Trajectory-level metrics cannot directly demonstrate more reliable tracking. Sequence 1 contains approximately 20s of challenging low-illumination segments where the exposure time is set to 20ms. It also exhibits larger illumination variations than Sequence 2, making it the sequence where risk classification and adaptive behavior are most fully manifested. In this section, frames are divided into low, medium, and high risk levels according to the three quantiles of $q_{k}$ output by A2 (Equation (13)). Under the complete front-end configuration, the feature-level metrics listed in Table 10 are statistically analyzed across different risk levels to verify the capability of risk classification in characterizing feature quality.

All three core reliability metrics exhibit monotonic degradation from low to medium and high risk levels. The inlier ratio after filtering decreases from 80.5% to 55.6%, the average tracking length decreases from 53.3 frames to 10.4 frames, and the feature survival rate decreases from 80.4% to 54.8%. Meanwhile, the proportions of features removed by saturation and ZNCC rejection increase accordingly, which is consistent with the physical interpretation of the risk components described in Section 3.4.1. The correlation coefficient between $q_{k}$ and the frame-wise inlier ratio is 0.587, indicating that the frame-wise risk score provides cross-frame explanatory capability for feature quality and supports the rationale of using $q_{k}$ to drive adaptive thresholds.

These feature-level results serve as a validation of the effectiveness of risk classification. The benefit of adaptive processing for high-risk frames is reflected in the trajectory-level accuracy improvements in Section 4.2.2 and the computational savings in Section 4.4. Considering single-frame statistics, the two front ends exhibit similar performances.

### 4.3. Parameter Settings and Robustness Analysis

The risk model and adaptive front end involve a set of normalized reference values, weights, and thresholds. Table 11 summarizes all parameter settings. The reference values, weights, and thresholds are first determined using the two indoor cart sequences in Section 4.1 with trajectory accuracy as the optimization objective. These parameters are then directly transferred to the two sequences in Section 4.2 without further tuning. Therefore, the ablation study in Section 4.2 can also be used to validate parameter transferability.

All adaptive quantities are constrained by explicit bounds. The GFTT quality threshold is limited to 0.01–0.04, the KLT search window is restricted to three discrete levels of 21, 25, and 31, and the forward–backward threshold is bounded within 0.5–1.2. Therefore, the influence of each parameter on the output is bounded, and no unbounded sensitivity direction exists. The complete front end still reduces the ATE RMSE by 16.8% and 12.9%, respectively, providing a natural robustness evaluation under unchanged parameters and substantially different conditions.

The isolation study presented in Section 4.2.2 further provides a performance bound under parameter mismatch. When the frame-wise risk is fixed to constant values and all adaptive thresholds degenerate into fixed thresholds, the ATE RMSE of the two sequences increases by 12.5% and 6.6%, respectively. The system remains operational in both cases, indicating that the impact of parameter deviation is bounded and moderate. A comprehensive parameter-wise sensitivity analysis is left for future work, and this limitation is explicitly acknowledged in the Section 5.

### 4.4. Computational Overhead of the Adaptive Front End

The additional computational cost is measured on the same platform and the same data sequence, and compared with the baseline front end. On Sequence 1, the baseline and complete front end are each executed once with identical input frames (3730 frames with frame-wise alignment). The average per-frame runtime of each component is recorded through the internal timing mechanism of the front end. The front end runs in a single-threaded mode, and the measurement platform is equipped with an AMD Ryzen 9 9950X CPU at 3000MHz. The final results are presented in Table 12.

Among the additional components, the A2 risk computation and adaptive parameter adjustment introduce only 0.005ms and 0.001ms per frame, respectively, which are at the scale of scalar operations and table lookup. The A5 rotation prediction is below the timing resolution of the measurement. The main additional cost comes from the A3 patch-level filtering, which introduces 0.329ms per frame and increases linearly with the number of candidate points and patch size.

The baseline front end requires 18.148ms per frame, while the complete front end requires only 17.068ms per frame, which is approximately 1.080ms lower than the baseline. This reduction is attributed to the adaptive increase in the quality threshold, which results in fewer features being detected and tracked in high-risk frames. Consequently, the feature detection time decreases from 11.708ms to 10.437ms, and the saved computation exceeds the additional overhead introduced by the adaptive modules. The average processing rates are 55.102Hz and 58.589Hz for the baseline and complete front end, respectively. Both are approximately 2.2 to 2.3 times the camera frame rate of 25Hz, demonstrating that the front end maintains real-time performance without increasing the overall computational burden. It should be noted that the runtime is measured on a desktop-class CPU. Nevertheless, the additional overhead introduced by the adaptive modules is only 0.335ms per frame, i.e., about 2% of the per-frame processing budget, and the total per-frame runtime of 17.068ms is well below the 40ms frame interval, indicating that the proposed front end is fundamentally compatible with resource-constrained embedded platforms. Dedicated embedded profiling and optimization, as well as validation on public datasets and additional platforms, are left for future work.

## 5. Conclusions

This paper presented HS-ES-VINS, a hardware-synchronized and exposure-state-aware extension of VINS-Fusion for stereo VIO on industrial platforms operating under auto-exposure, auto-gain, and dynamic motion. The proposed framework compensates for camera–IMU temporal misalignment at two levels. At the hardware level, the effective sampling time of each global-shutter image is reconstructed at the exposure midpoint, which removes the frame-dependent exposure component of the offset that neither fixed nor online scalar compensation can remove: under 20 ms low-light exposure, referencing the frame timestamp to the exposure midpoint reduces the ATE RMSE by 28.6% and 22.7% relative to the exposure-start timestamp under fixed and online offset compensation, respectively. The residual error is reduced to identifiable constant terms—the IMU-side output delay (13.2 ms for the gyroscope channel of the STIM300) and sub-millisecond camera-side reporting residuals—which are absorbed equivalently by fixed Kalibr compensation (13.828 ms) and online estimation (approximately 15.0 ms), as cross-validated by three independent sources of evidence. At the front-end level, the exposure–gain–motion risk model drives adaptive feature detection, KLT tracking, correspondence filtering, and IMU rotation prediction. The complete front end reduces the ATE RMSE by 16.8% and 12.9% and the ATE standard deviation by 21.2% and 12.8% on indoor multi-floor and outdoor cycling sequences, respectively, and the feature-level statistics confirm that the frame-wise risk score explains the variation in tracking quality. The complete front end requires 17.1 ms per frame, which is less than the baseline, so these improvements are obtained without additional computational burden.

Several limitations of this work should be noted. First, the motion-blur risk model considers rotational motion only; translational blur is depth-dependent, and rolling-shutter cameras would require row-wise exposure-midpoint modeling, so extension to other camera types and aggressive translational maneuvers is left for future work. Second, the risk-model parameters and adaptive thresholds are empirically set within explicit bounds; the parameter transfer and isolation experiments indicate moderate and bounded sensitivity, but a comprehensive parameter-wise sensitivity analysis remains to be conducted. Third, the evaluation covers self-collected indoor and outdoor sequences on a single hardware platform with independent ground truth; validation on public datasets, additional sensor platforms, and embedded real-time hardware is required before broader industrial deployment, since visual-only estimation remains vulnerable to dynamic illumination changes, motion blur, and aggressive motions [2]. In summary, the proposed method reduces and compensates for the frame-dependent component of temporal misalignment rather than eliminating temporal errors entirely, and the remaining constant residuals are absorbed by the estimator.

## Figures and Tables

**Table 1:** Calculation of total delay (non-triggered mode).

Delay Component (ms)	Gyroscope	Accelerometer (10 g)
$t_{\mathrm{Sensor}\&\mathrm{ADC}}$	0.71	5.19
Low-pass Filter Delay	12.00	12.00
$t_{\mathrm{LPF}\_\mathrm{DelayOffset}}$	−0.05	−0.19
Optional Delay	0	—
Output Delay	0.50	0.50
$t_{\mathrm{tov}\_\mathrm{dl}}$	0.0012	0.0012
$t_{\mathrm{tx}\_\mathrm{dl}}$ (max)	0.08	0.08
Serial Transmission	0.640	0.640
Total Delay	≈13.9	≈18.2

Note: The total output delays of each channel in non-triggered mode are calculated according to Equation (2). Since MS-HTSS records the timestamp at the beginning of the STIM300 data packet reception, the serial transmission delay term is excluded. Therefore, the total delay of the gyroscope channel is approximately 13.2 ms, while that of the accelerometer channel is approximately 17.6 ms.

**Table 2:** RMSE and STD results for ATE and RPE in low-light indoor corridor scenario (unit: m).

Timestamp Type	Method	ATE		RPE		Time Offset (ms)
		RMSE	STD	RMSE	STD	
Exposure Start	Kalibr	0.607640	0.372803	0.065984	0.022089	13.828
	Online Estimation	0.536805	0.315581	0.065003	0.019544	23.152
Exposure Midpoint	Kalibr	0.434001	0.197789	0.063485	0.019278	13.828
	Online Estimation	0.415141	0.186311	0.064795	0.019026	14.979
Exposure End	Kalibr	0.498214	0.235707	0.064103	0.018859	13.828
	Online Estimation	0.513622	0.243651	0.063858	0.018376	6.921

Note: Online estimation refers to the online time offset estimation implemented via VINS-Fusion, with values reported as the mean.

**Table 3:** RMSE and STD results for ATE and RPE in normal-light indoor corridor scenario (unit: m).

Timestamp Type	Method	ATE		RPE		Time Offset (ms)
		RMSE	STD	RMSE	STD	
Exposure Start	Kalibr	0.422579	0.208476	0.044431	0.011528	13.828
	Online Estimation	0.400713	0.198221	0.044895	0.011726	15.763
Exposure Midpoint	Kalibr	0.402471	0.197915	0.044908	0.011742	13.828
	Online Estimation	0.393125	0.192724	0.044820	0.011670	15.045
Exposure End	Kalibr	0.395994	0.191963	0.045016	0.011787	13.828
	Online Estimation	0.387660	0.188672	0.044864	0.011654	14.333

Note: Online estimation refers to the online time offset estimation implemented via VINS-Fusion, with values reported as the mean.

**Table 4:** ATE RMSE across timestamp reference and offset strategy in the controlled experiment (unit: m).

Illumination	Frame Timestamp Reference Point	Fixed Offset	Online Estimation
Low-Light (Constant Exposure 20 ms)	Exposure Start	0.608 (+40.0%)	0.537 (+29.3%)
	Exposure Midpoint (Ours)	0.434	0.415
	Exposure End	0.498 (+14.8%)	0.514 (+23.7%)
Normal-Light (Auto-Exposure 0.4 ms–6.4 ms)	Exposure Start	0.423 (+5.0%)	0.401 (+1.9%)
	Exposure Midpoint (Ours)	0.402	0.393
	Exposure End	0.396 (−1.6%)	0.388 (−1.4%)

Note: Values in parentheses denote the relative change compared to the midpoint row in the same column; a negative sign indicates better performance than the midpoint row. The Fixed Offset column uses the same Kalibr calibration value of 13.828 ms across all reference points.

**Table 5:** Modules of the adaptive front end.

Code	Module and Function	Role in Ablation
A1	Exposure-midpoint temporal alignment	Directly modifies output; corresponds to the first ablation row
A2	EGM risk evaluation	Serves as a driving signal; does not directly modify output. Provides per-frame input to the adaptive threshold of A3, as well as to A4 and A5; not assigned a standalone ablation row
A3	Local photometric consistency filtering	Directly rejects unreliable correspondences; the fixed-threshold variant does not depend on A2
A4	Adaptive feature extraction and tracking	Directly modifies output; the primary module through which the value of A2 is realized
A5	IMU rotation prediction	Directly modifies output (initial value only)

**Table 6:** Summary of the two experimental sequences used in the ablation study.

Sequence	Acquisition Method	Ground Truth	Duration (s)	Trajectory Length (m)	Mean Linear Velocity (m/s)	Mean Angular Velocity (rad/s)
Seq. 1	Backpack-mounted walking, closed-loop trajectory across floors	Faster-LIO	149.0	189.773	1.274	−0.004159
Seq. 2	Cycling, campus internal roads	RTK	118.881	926.808	7.796	−0.021346

**Table 7:** Ablation results of each module on the indoor multi-floor corridor walking sequence (unit: m).

Configuration	ATE		RPE	
	RMSE	STD	RMSE	STD
Baseline (Kalibr)	1.425739	0.786794	0.066635	0.019174
A1	1.401671	0.737919	0.060092	0.021995
A1 + A3	1.270780	0.675981	0.066880	0.022228
A1 + A3 + A4	1.245484	0.658551	0.065544	0.020833
A1 + A3 + A4 + A5	1.186760	0.620221	0.068398	0.023156

**Table 8:** Ablation results of each module on the campus internal road sequence (unit: m).

Configuration	ATE		RPE	
	RMSE	STD	RMSE	STD
Baseline (Kalibr)	6.482093	3.673430	1.974052	1.588388
A1	6.441036	3.386003	1.874798	1.461111
A1 + A3	5.991880	3.486874	1.962974	1.604044
A1 + A3 + A4	5.815951	3.317090	2.150282	1.770419
A1 + A3 + A4 + A5	5.646614	3.204901	2.007656	1.647214

**Table 9:** Ablation comparison of the EGM risk module (unit: m).

Sequence	Configuration	ATE		RPE	
		RMSE	STD	RMSE	STD
Seq. 1	A2 Enabled	1.186760	0.620221	0.068398	0.023156
	A2 Disabled	1.334665	0.713444	0.071942	0.021947
Seq. 2	A2 Enabled	5.646614	3.204901	2.007656	1.647214
	A2 Disabled	6.020970	3.511095	1.830369	1.452630

**Table 10:** Feature tracking statistics under different EGM risk levels.

Metric	Low-Risk Frames	Medium-Risk Frames	High-Risk Frames
Mean Number of Successfully Tracked Features	150.0	144.2	128.6
Inlier Ratio after Filtering (%)	80.5	71.2	55.6
Mean Feature Track Length (frames)	53.3	26.1	10.4
Feature Survival Rate (%)	80.4	70.9	54.8
Forward–Backward Error (median, px)	0.004	0.007	0.240
Saturation Rejection Ratio (%)	12.6	17.7	18.6
ZNCC Rejection Ratio (%)	0.7	0.9	2.8
LK Residual Rejection Ratio (%)	0.0	0.0	0.9

Note: Results are obtained with the full front end on Seq. 1 (3730 frames). Risk bins are divided by the tertiles of $q_{k}$ at 0.798/0.913.

**Table 11:** Parameters of the EGM risk model and the adaptive front end.

Category	Parameter	Meaning	Value
Normalization References	$t_{\mathrm{ref}}$, $G_{\mathrm{ref}}$, $B_{\mathrm{ref}}$, $\Delta A_{\mathrm{ref}}$	Exposure duration/gain (linear, log-normalized)/pixel blur references/Reference inter-frame exposure–gain variation (natural-log units)	10 ms, 8.0, 2 px, 1.0
Numerical Stabilizers	$\epsilon_{t}$, $\epsilon_{g}$	Log-stabilizing constants in Equation (5)	$1\times 10^{-9}$
Risk Weights	$w_{1}$–$w_{4}$	Exposure/gain/change/motion blur weights	0.25/0.25/0.20/0.30
Risk Normalization	—	Truncation range of each risk component	[0, 3]
Feature Extraction	$Q_{\mathrm{GFTT}}$	GFTT quality threshold	$\mathrm{clip}\left(0.01+0.01\cdot (r_{\mathrm{gain}}+r_{\mathrm{change}}+r_{\mathrm{motion}}),\;0.01,\;0.04\right)$
KLT Window	$W_{\mathrm{LK}}$	Search window (segmented by $B_{k}$)	21/25/31 ($B_{k}\leq 2$, $2<B_{k}\leq 4$, $B_{k}>4$)
Pyramid	$L_{\mathrm{pyr}}$	Number of levels	3/4 (4 levels when $\theta_{\mathrm{frame}}>0.03$)
FB Threshold	$\tau_{\mathrm{fb}}$	Forward–backward threshold	$\mathrm{clip}(0.5+0.3r_{\mathrm{motion}},\;0.5,\;1.2)$
Photometric Consistency Filtering	patch size/$T_{\mathrm{sat}}$	Patch side length for saturation/ZNCC/LK checks; saturation threshold (8-bit)	11×11 px/245
Photometric Consistency Filtering	$\tau_{s}$, $\tau_{\mathrm{zncc}}$, $\tau_{\mathrm{LK}}$	Saturation ratio gate/ZNCC gate/LK photometric residual threshold	0.15, 0.65, $\mathrm{clip}(30q_{k},8,\;30)$
Rotation Prediction	—	Activation condition	$\theta_{\mathrm{frame}}>0.01$ and $B_{k}>1$ px
Baseline Offset	—	Kalibr fixed offset (shared across sequences)	13.828 ms

**Table 12:** Per-frame runtime and total processing time of each front-end stage (unit: ms).

Stage	Baseline Front End	Full Adaptive Front End
Feature Detection (GFTT)	11.708	10.437
Optical Flow Tracking (KLT)	1.199	1.180
Stereo Matching	2.545	2.461
A2 Risk Computation	—	0.005
A4 Adaptive Parameter Adjustment	—	0.001
A3 Patch-Level Filtering (Saturation/ZNCC/LK)	—	0.329
A5 Rotation Prediction	—	0.000
Others (Feature Management, Debug Statistics, etc.)	2.696	2.655
Total Per-Frame Runtime	18.148	17.068
Average Processing Frame Rate (Hz)	55.102	58.589

## Data Availability

The data presented in this study are available on request from the corresponding author.
